# Major Cat Allergen Fel d 4: Structure and Identification of a Cross‐Reactive IgE‐Epitope‐Containing Area

**DOI:** 10.1111/all.70146

**Published:** 2025-11-24

**Authors:** Nikolina Todorović, Daria Trifonova, Zicheng Liu, Mirela Curin, Laszlo Schooltink, Theo Sagmeister, Christoph Grininger, Renata Kiss, Nina Gottstein, Bernd Gesslbauer, Andreas Winkler, Tea Pavkov‐Keller, Alexander Karaulov, Rudolf Valenta, Walter Keller

**Affiliations:** ^1^ Institute of Molecular Biosciences University of Graz Graz Austria; ^2^ Division of Immunopathology, Department of Pathophysiology and Allergy Research, Center for Pathophysiology, Infectiology and Immunology Medical University of Vienna Vienna Austria; ^3^ Laboratory for Immunopathology, Department of Clinical Immunology and Allergology Sechenov First Moscow State Medical University Moscow Russia; ^4^ Life Improvement by Future Technologies (LIFT) Center, Skolkovo Moscow Russia; ^5^ Institute of Pharmaceutical Sciences University of Graz Graz Austria; ^6^ Institute of Biochemistry Graz University of Technology Graz Austria; ^7^ Biotechmed‐Graz University of Graz Graz Austria; ^8^ Field of Excellence Biohealth University of Graz Graz Austria; ^9^ Center for Molecular Allergology Karl Landsteiner University of Health Sciences Krems Austria

**Keywords:** cat allergy, cross‐reactivity, crystal structure, epitope, Fel d 4

## Abstract

**Background:**

Allergic sensitization to cats and other furry animals is a major cause of asthma and allergic rhinitis in more than 200 million people worldwide. According to the frequency of IgE recognition, allergen‐specific IgE levels, and allergenic activity, Fel d 4 is a major allergen in the cat (*Felis domesticus*). The lipocalin allergen Fel d 4 is highly homologous to dog (Can f 6) and major horse (Equ c 1) allergens. Accordingly, IgE cross‐reactivity to these allergens contributes to polysensitization and allergic responses upon exposure to different animals.

**Methods:**

Fel d 4 was recombinantly produced in two systems, *E. coli* and Expi293F mammalian cells. Recombinant forms were characterized by circular dichroism and mass spectrometry. The Fel d 4 3D structure was determined using X‐ray crystallography. Immunoreactivity, epitope analyses, and cross‐reactive properties were assessed by ELISA and basophil release assays using allergic patients’ sera.

**Results:**

We reveal the rFel d 4 crystal structures and demonstrate that mammalian cells produce an N‐glycosylated recombinant Fel d 4 allergen. The C‐terminal regions of Fel d 4, Can f 6, and Equ c 1 constitute conformational IgE‐epitope‐containing areas responsible for cross‐reactivity.

**Conclusion:**

Uncovering the IgE‐binding sites of Fel d 4 and cross‐reactive allergens contributes to future rational design of active and passive allergen‐specific treatment forms.

## Graphical abstract

- This study reports the three‐dimensional structure of an important cat allergen, Fel d 4, that shares high homology with Equ c 1 (i.e., major horse allergen) and Can f 6 (i.e., dog allergen).
- The C‐terminal region of the Fel d 4 contains a major IgE‐reactive epitope.
- The C‐terminal epitope‐containing area plays a role in the cross‐reactivity between Fel d 4, Equ c 1, and Can f 6.

Abbreviations: ELISA, enzyme‐linked immunosorbent assay; IgE, immunoglobulin E; OD, optical density; PDB, Protein Data Bank; RBL, rat basophil leukemia; sIgG, specific IgG.

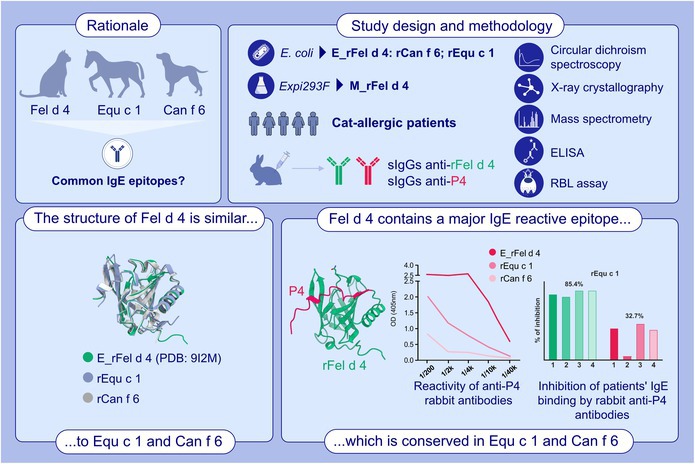

**Abbreviations**
AIT: allergen immunotherapy
CD: circular dichroism
IgE: immunoglobulin E
LC–MS/MS: liquid chromatography‐coupled tandem mass spectrometry
OD: optical density
PDB: Protein Data Bank
PTM: posttranslational modification
RMSD: root‐mean‐square deviation
sfGFP: superfolder green fluorescent protein
TEV: tobacco etch virus

## 1 Introduction

Pet allergy has become a significant health concern in today's society, with pets being a significant source of indoor allergens [1, 2, 3]. Immunoglobulin E (IgE)‐mediated hypersensitivity toward furry animals, in particular cats, can lead to respiratory symptoms, such as rhinoconjunctivitis and severe asthma [4, 5, 6]. Among pet allergens reported within the WHO/IUIS database (allergen.org), eight allergens have been recognized in the domestic cat (*Felis domesticus*), designated as Fel d 1 to 8. Although Fel d 1 is the major, most extensively studied, and cat‐specific allergen [4, 7, 8, 9, 10], recent reports have highlighted the significant clinical relevance of other cat allergens. When assessing IgE‐recognition frequency among cat‐allergic patients, it has been shown that Fel d 3, Fel d 4, Fel d 7, and Fel d 8 are additional relevant cat allergens [11, 12]. According to allergen‐specific IgE levels and allergenic activity evaluated by in vitro basophil degranulation experiments, Fel d 4 and Fel d 7 [13] stood out, suggesting that they should be included in diagnostic approaches and allergen‐specific forms of treatment.

Fel d 4 is among the most frequent sensitizing cat allergens, and according to reports, up to 63% of cat‐allergic individuals have Fel d 4‐specific IgE [13, 14]. Its involvement in multi‐sensitization in cat allergy was found to correlate with the extent and severity of respiratory diseases. Co‐sensitization toward Fel d 4 and Fel d 1 was frequently observed in asthmatic patients and is associated with an increased risk of asthma, wheezing, and atopic dermatitis in school children [6, 15, 16, 17].

Allergens of the lipocalin protein family share, on average, low sequence identity despite high structural similarity. Fel d 4 is a lipocalin and is of high interest because it has been shown to share IgE epitopes with homologous allergens in dogs (i.e., Can f 6) and in horses (i.e., Equ c 1). Accordingly, Fel d 4‐allergic patients often suffer from allergic symptoms upon contact with dogs and horses due to IgE cross‐reactivity [18, 19, 20, 21]. Clinically relevant cross‐reactivity was confirmed with IgE‐inhibition studies using cat‐ and dog‐allergic patient serum [18, 20, 22]. Furthermore, guinea pig lipocalin allergen (Cav p 6), with 54% sequence identity to Fel d 4, showed cross‐reactivity to Fel d 4 and Can f 6 [23]. Cross‐reactivity of Fel d 4 with the major allergens from mice, Mus m 1, and rats, Rat n 1, is very likely due to the relatively high sequence identity of approximately 55% [24, 25, 26]. Despite a low sequence identity of 22%, patient‐dependent cross‐reactivity with dog lipocalin allergen Can f 2 was detected for certain patients with high levels of Fel d 4‐specific IgE [27]. Hence, sequence and structure‐based analysis supported by IgE‐inhibition studies using allergic patients' sera are crucial for reasoning possible allergen‐specific and cross‐reactive B‐cell epitopes recognized by IgE.

Here, we recombinantly produced cat allergen Fel d 4 using two different expression systems, *E. coli* and Expi293F, a mammalian cell line, compared their biophysical profiles, and obtained a high‐resolution X‐ray crystal structure of Fel d 4. Using high‐resolution native MS and LC–MS/MS, we revealed glycosylation properties of the two recombinant allergens. Importantly, we were able to locate a major IgE epitope‐containing area of Fel d 4 responsible for cross‐reactivity with Can f 6 and Equ c 1 in the C‐terminal portion of Fel d 4. Our findings contribute to the understanding of the structural properties of cat allergen Fel d 4 and define the position of the main cross‐reactive IgE epitope, with important implications for the development of molecular allergy tests and molecular allergy vaccines.

## 2 Materials and Methods

### 2.1 Expression and Purification of Recombinant Fel d 4

The coding sequence for Fel d 4 (GenBank AY497902) without the N‐terminal signal sequence and with a N‐terminal TEV protease cleavable hexa‐histidine tag was cloned into the pET‐27b vector (Novagen, USA) for recombinant production in *E. coli* SHuffle T7 Express *lysY* cells (New England Biolabs, USA), affinity and analytical purification as described in Supporting Information.

For transient eukaryotic protein expression, the gene encoding mature Fel d 4 was cloned into pcDNA 3.4 TOPO vector (ThermoFisher Scientific, USA), and the final construct harbored an N‐terminal signal sequence (UniProt P01661) followed by TEV‐cleavable sfGFP and a hexa‐histidine tag. Eukaryotic expression in Expi293F cells (ThermoFisher Scientific, USA) and protein purification were performed as described in Supporting Information. To determine secondary structure and temperature stability, purified proteins were analyzed with circular dichroism spectroscopy. A detailed description of the methods is provided in the online form in Supporting Information.

### 2.2 Intact Mass Spectrometry

Aliquots of 3 μL of protein preparations at 10 μM were desalted on a Shim‐pack Scepter C4‐300 (G) column (3 μM) (Shimadzu, Japan) by washing with a solution of 1% acetonitrile and 0.1% formic acid. A gradient of acetonitrile (1%–95%) eluted the proteins into an Impact II ESI‐Q‐TOF (Bruker, USA) mass spectrometer. Protein signatures in the mass spectra were integrated and deconvoluted using the maximum entropy function of DataAnalysis (Bruker, USA).

### 2.3 Liquid Chromatography‐Mass Spectrometry (LC–MS/MS) Analysis

Protein samples were diluted with 50 mM NH_4_HCO_3_ to a final concentration of 0.3 mg/mL. Proteins were reduced with 10 mM dithiothreitol at 56°C for 30 min, alkylated with 20 mM iodoacetamide for 20 min, and subjected to 16 h digestion with trypsin (sequencing grade, Promega, USA). LC–MS/MS analysis of peptides was performed on an UltiMate 3000 RSLCnano system in line with a LTQ XL mass spectrometer (ThermoFisher Scientific, USA) as reported [28]. LTQ‐XL data were analyzed with Mascot (Matrix Science, UK) and a fasta file containing rFel d 4 protein sequences. Results were filtered based on peptide scores ≥ 25, and a 1% false discovery rate was applied using Mascot.

### 2.4 Crystallization and X‐Ray Diffraction Data Collection

Crystallization experiments were performed with an Oryx8 robot (Douglas Instruments Limited, UK) as described in Supporting Information. Collected data were processed using the XDS [29] program package and scaled and merged using AIMLESS [30].

### 2.5 Structure Determination, Model Building, and Refinement

Structures were solved by molecular replacement via Phaser [31]. The AlphaFold2 [32] model of Fel d 4 was used as an initial search model for M_rFel d 4, whereas M_rFel d 4 was used for the structure determination of E_rFel d 4. Alternating model building and refinement were performed in Coot [33], Refmac [34] within the CCP4i2 program suite [35, 36], and phenix.refine [37]. The stereochemistry and geometry were analyzed using MolProbity [38]. A summary of data processing and refinement statistics is provided in Table 1. Atomic coordinates and structure factors have been deposited in the Protein Data Bank under accession codes 9I2M (E_rFel d 4) and 8AMC (M_rFel d 4). All structure figures were prepared in ChimeraX [39] and PyMOL (The PyMOL Molecular Graphics System, Version 3.0 Schrödinger LLC).

**TABLE 1:** Data collection and refinement statistics.

	E_rFel d 4	M_rFel d 4
PDB accession code	9I2M	8AMC
Data collection		
Space group	*I 2 2 2*	*P 2*_*1*_
Cell dimensions		
a, b, c (Å)	64.67, 77.91, 79.15	67.29, 120.57, 67.15
α, β, γ (°)	90.00, 90.00, 90.00	90.00, 106.51, 90.00
Resolution (Å)	38.96–1.55 (1.55–1.605)	34.09–2.95 (2.95–3.055)
No. of unique reflections	29,388 (2911)	21,621 (2177)
R_merge_	0.0604 (0.8949)	0.1545 (1.075)
CC1/2	1 (0.878)	0.993 (0.662)
I/*σ*I	21.01 (3.13)	9.83 (2.06)
Completeness (%)	99.94 (99.97)	99.6 (100.00)
Redundancy	13.2 (13.6)	6.5 (6.8)
Refinement		
R_work_/R_free_	0.1730/0.2149	0.1907/0.2310
No. of atoms	1700	5247
Macromolecules	1517	5138
Ligand/ion	0	10
Water	183	99
Average B‐factor	28.80	67.11
R.m.s. deviations		
Bond lengths (Å)	0.010	0.015
Bond angles (°)	1.06	1.96
Ramachandran plot (%)		
Favored/allowed/disallowed	98.28/1.72/0.00	94.69/5.31/0.00

*Note:* Values in parentheses are for the highest‐resolution shell.

### 2.6 Sera From Cat Allergic Patients

Serum samples of cat‐allergic patients and nonallergic subjects were obtained at the Department of Pathophysiology and Allergy Research, Medical University of Vienna, with the approval of the Ethics Committee of the Medical University of Vienna (EK 1641/2014). For all participants signed informed consent was obtained. All patients were well characterized in terms of clinical symptoms collected using the adapted ISAAC questionnaire [40], including demographic data as well as symptoms upon cat contact. Specific IgE levels to the Fel d 4 allergen were measured by ImmunoCAP (ThermoFisher Scientific, Sweden). IgE levels ≥ 0.1 kUA/L were considered positive [41]. The demographic and clinical characteristics of participants are presented in Table S19. Four Equ c 1‐sensitized patients included in the IgE competition ELISA experiments, that is, patients 9, 11, 13, and 21, also exhibited IgE reactivity to Fel d 1 (9: 20.6 kUA/L; 11: 7.39 kUA/L; 13: 19.5 kUA/L; 21: 20.6 kUA/L). IgE reactivity toward rFel d 4 allergens (i.e., M_rFel d 4, E_rFel d 4, and deglycosylated M_rFel d 4 PNGase F) was assessed through direct ELISA using additional cat allergic patients containing Fel d 4‐specific IgE antibodies and sera from nonallergic subjects (Tables S9, S10). The allergenic activity was analyzed using the Rat Basophil Leukemia (RBL) assay. Rabbit antisera specific for E_rFel d 4 and Fel d 4‐derived peptide 4 were obtained as described [42], and the ability of the rabbit antisera to inhibit IgE binding to Fel d 4 and cross‐reactive allergens was analyzed in IgE competition ELISA experiments. Detailed methodology is provided in Supporting Information.

### 2.7 Statistical Analysis and Visualization Tools

The statistical program SPSS (2008; version 16.0 SPSS Inc., USA), GraphPad Prism 6 software (GraphPad Software, USA), and OriginPro 2023b (OriginLab Corporation, USA) were used for statistical analysis and figure preparation. *p* < 0.05 were considered significant. Differences between groups were compared by Student's dependent t‐test for paired samples and the Wilcoxon matched‐pairs test.

## 3 Results

### 3.1 Biophysical Properties of Recombinant Fel d 4 From Two Expression Systems Are in Agreement

**FIGURE 1:**
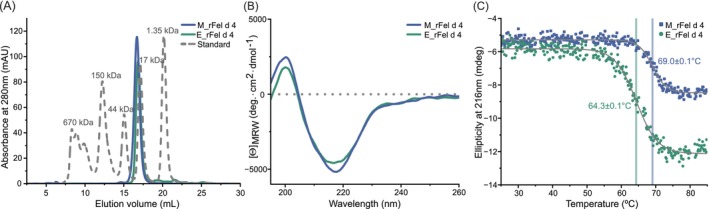
Comparative overview of recombinant M_rFel d 4 and E_rFel d 4. (A) Analytical size‐exclusion chromatography recorded at 280 nm for the M_rFel d 4 (blue line) and E_rFel d 4 (green line) superimposed to a gel filtration standard (gray dashed line; Thyroglobulin (bovine)‐670 kDa, γ‐globulin (bovine)‐158 kDa, Ovalbumin (chicken)‐44 kDa; Myoglobin (horse)‐17 kDa, Vitamin B12‐1.35 kDa) (Bio‐Rad, CA, USA). (B) CD analysis of M_rFel d 4 (blue) and E_rFel d 4 (green). The mean residue ellipticities (y‐axis: Degree·cm^2^·dmol^−1^) were recorded in the wavelength range from 195 to 260 nm (x‐axis). (C) Ellipticity recorded at 216 nm (y‐axis: mdeg) as a function of temperature (x‐axis: °C) for M_rFel d 4 (blue) and E_rFel d 4 (green).

To investigate the biophysical and immunological properties of the glycosylated and nonglycosylated forms of Fel d 4, we produced the cat allergen Fel d 4 in a prokaryotic ( *Escherichia coli* ), that is, E_rFel d 4, and in a eukaryotic host (Expi293F cells), that is, M_rFel d 4. Both proteins were expressed with an N‐terminal hexa‐histidine tag and purified by Ni‐affinity chromatography. The tag was cleaved off only in M_rFel d 4. A final step of analytical size‐exclusion chromatography was applied (Figure 1A), resulting in highly pure monomeric allergens (Figure S1A).

Both recombinant proteins were produced folded, exhibiting very similar CD spectra indicative of a typical lipocalin fold predominantly composed of β‐sheets (Figure 1B). A deglycosylated form of M_rFel d 4 (i.e., M_rFel d 4 PNGase F) obtained by PNGase F treatment also showed a similar fold as compared to M_rFel d 4 and E_rFel d 4 (data not shown). Thermal unfolding analysis proved the high stability of E_rFel d 4 and M_rFel d 4, with a Tm of 64.3°C ± 0.1°C for E_rFel d 4°C and 69.0°C ± 0.1°C for M_rFel d 4 (Figure 1C). When heated to 95°C, both allergens exhibited a minimum at 205 nm, indicating partial unfolding upon heating (Figure S2). Significant alterations in the spectral features compared to the spectrum before heating developed when proteins were gradually cooled to 20°C. These observations demonstrate that the unfolding of the two recombinant Fel d 4 allergens by heating to 95°C is irreversible.

### 3.2 Cat Allergen rFel d 4 Displays a Lipocalin Fold

**FIGURE 2:**
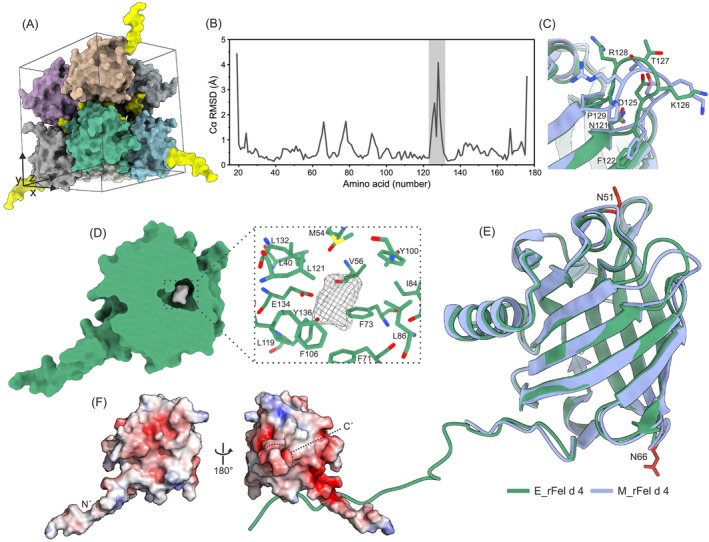
Properties of M_rFel d 4 and E_rFel d 4. (A) Unit cell within crystal lattice of the E_rFel d 4 containing 8 protein monomers in surface representation. N‐terminal extensions contributing to crystal contact formation are colored yellow. (B) Cα RMSD (y‐axis) between E_rFel d 4 and M_rFel d 4 (chain A) plotted per amino acid residue (x‐axis). (C) Inset showing the loop and residue positions, in stick representation, with the highest Cα RMSD between the two recombinant allergens. (D) Surface representation of E_rFel d 4 3D structure and residual electron density (Fo‐Fc electron density map in gray mash, contoured at 2.5 RMSD) found within the cavity, with inset showing cavity residues in stick representation. (E) Superimposed crystal structures of M_rFel d 4 (blue, chain A, PDB: 8AMC) and E_rFel d 4 (green, PDB: 9I2M) in cartoon representation. (F) Electrostatic potential calculated with the adaptive Poisson‐Boltzmann solver (APBS) for the E_rFel d 4 structure in surface representation with positive and negative potential presented in blue (5 kT/e) to red (−5 kT/e) color gradient, respectively, (C′‐C terminus, N′‐N terminus).

The M_rFel d 4 structure (refined at 2.95 Å resolution in the space group of *P 2*_*1*_) (Table 1) contains four monomers (chains A to D) in the asymmetric unit (AU) with an average root mean square deviation (RMSD) of 0.409 Å between four chains. The independent molecules in the AU did not exhibit biological interfaces, but the interactions between them were dominated by crystal packing forces (PDBePISA) [43]. The E_rFel d 4 structure (refined at 1.55 Å resolution, space group *I 2 2 2*) (Table 1) exhibits one molecule in the AU. The crystal packing was greatly supported by the additional flexible but, in this case, stabilizing N‐terminal residues (Figure 2A). The presence of electron density allowed for the building of 11 amino acid residues at the N‐terminus containing a TEV cleavage site with a linker toward a histidine tag. The structural differences between the E_rFel d 4 and M_rFel d 4 3D models proved to be very low, with an average of 0.615 Å in Cα RMSD (Table S1). The highest deviations (Figure 2B) occurred in regions of flexible, surface‐exposed loops (Figure 2C), implying that the N‐terminal residue extension did not influence the original fold of the molecule (Figure 2E). As the two structures were closely matching, E_rFel d 4 was used for structural representations and residue numbering.

The observed structural architecture in Fel d 4 aligned well with the conserved lipocalin protein family fold [44], consisting of an 8‐stranded anti‐parallel β‐barrel with a centered ligand binding cavity, α‐helix followed by an additional small β‐strand and a 3_10_‐like helix (Figure 2E). The overall structure of Fel d 4 was stabilized by an intramolecular disulfide bridge between amino acids C81 and C171. This bridge of two secondary structure elements, largely conserved among lipocalins, secures the connection of the C‐terminal tail to the rest of the structure. The central core of the β‐barrel contained a cavity surrounded by mainly hydrophobic residues. The size of the E_rFel d 4 cavity was ~120 Å^3^ as calculated using CavitOmiX (v. 1.0, 2022, Innophore GmbH) [45]. In the cavity of the E_rFel d 4 crystal structure, we found residual electron density with an elongated and planar shape, suggesting the presence of a small aromatic molecule (Figure 2D, S3). The observed density could not be assigned to any of the compounds used during protein purification or crystallization; therefore, we assumed that it originated from the bacterial expression system. In the 3D model of M_rFel d 4, the cavity was only partially occupied, with electron density in two out of four monomers of the AU. In this case, the density volume was smaller and modeled using water molecules. Importantly, the alignment of monomers and RMSD values (Table S1) suggests that the tertiary structure of the E_rFel d 4 was not affected by ligand binding, as the surface‐exposed amino acid residues remain unchanged. Nevertheless, ligand binding in some allergens may affect the 3D structure and, therefore, the IgE binding may depend on the presence of the ligand as reported for the calcium‐binding allergen Bet v III [46] and for nsLTPs [47]. Mapping of the surface electrostatic charge in Fel d 4 showed a high prevalence of negative charge patches on the surface of the allergen (Figure 2F), which could potentially form hydrogen or electrostatic interactions with specific paratopes of the IgE antibodies.

### 3.3 Oligosaccharides Occupy One N‐Glycosylation Site in M_rFel d 4

**FIGURE 3:**
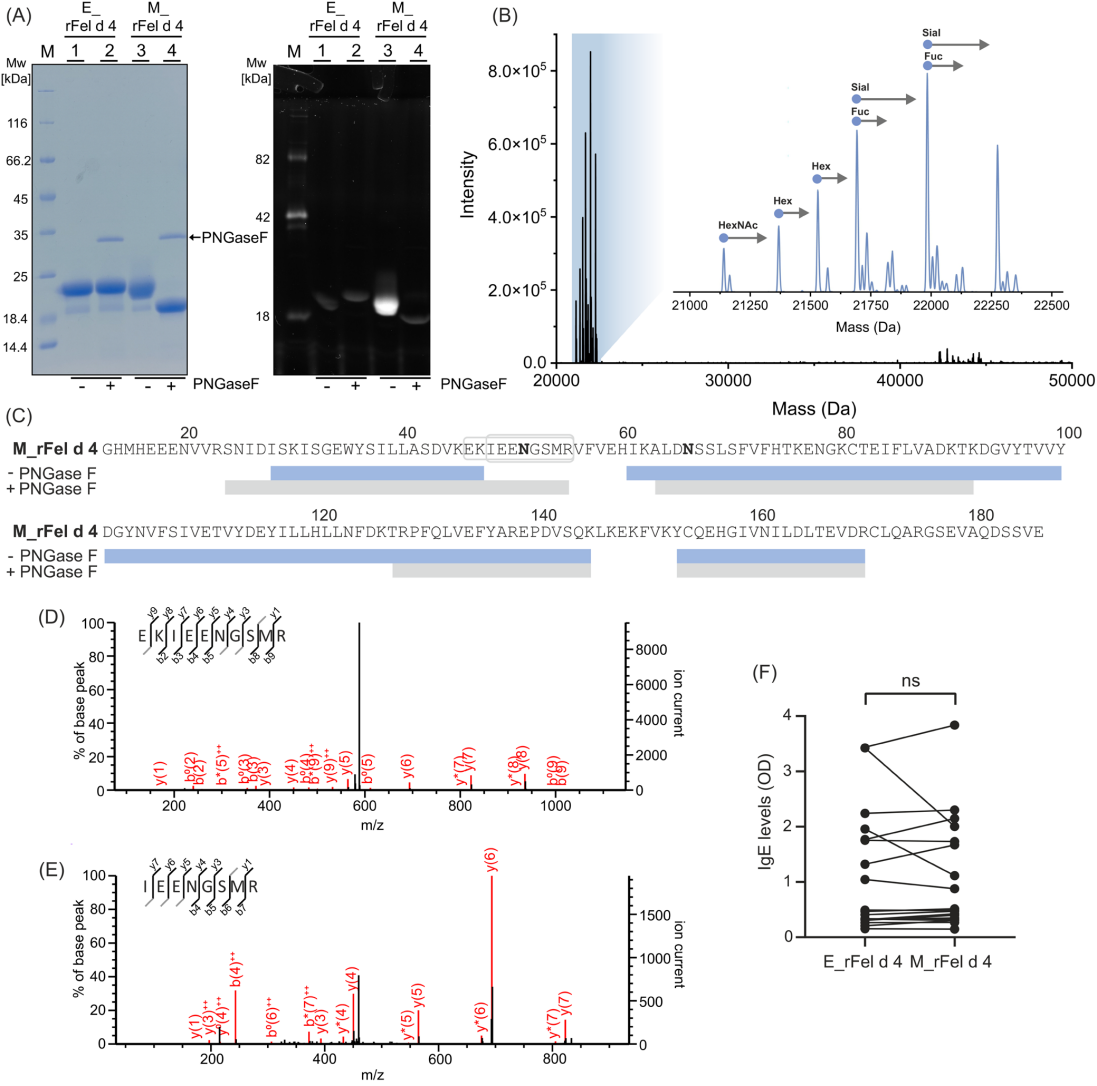
Glycosylation analysis and IgE recognition of E_rFel d 4 and M_rFel d 4. (A) SDS‐PAGE separation of E_rFel d 4 and M_rFel d 4 nontreated (lanes 1 and 3) and treated (lanes 2 and 4) with PNGase F, under denaturing conditions stained with Coomassie blue (left panel) and Pro Q Emerald 300 glycosylation staining (right panel). (B) Deconvoluted mass spectra of M_rFel d 4. The plot of intensity (y‐axis) versus mass (x‐axis: Da) with the inset into the region of interest shows the microheterogeneity of the glycosylated M_rFel d 4. Mass differences between major peaks are indicated with suggested glycan moieties. Hex, hexose; HexNAc, N‐acetylhexosamine; Fuc, fucose; Sial, sialic acid. (C) Sequence coverage for PNGase F‐treated M_rFel d 4 and M_rFel d 4. Matched peptides are shown below sequence in blue (M_rFel d 4) and gray (M_rFel d 4 PNGase F treated). (D, E) b and y ion distribution in MS/MS fragmentation spectrum for N51 containing peptides. Individual b and y ion m/z values for peptides are presented in Tables S4, S5. (F) Comparison of IgE reactivity of the two recombinant Fel d 4 proteins. Average OD values (405/492 nm, y‐axis) correspond to bound specific IgE for 19 cat‐allergic patients as measured by ELISA. Dots show the individual values for each patient. The statistical difference was assessed using the Students' t‐test and is shown above, a *p* value < 0.05 was considered statistically significant.

Lipocalin allergens have varying glycosylation properties, where some carry N‐ and/or O‐glycosylation and others are nonglycosylated [48]. Using NetNGlyc 1.0 [49], we identified two putative glycosylation sites on Fel d 4, N51 and N66, consistent with previous reports [14]. They are located within surface‐exposed loop regions and surface‐exposed, suggesting their accessibility for modification (Figure 2E). As prokaryotic and eukaryotic expression systems have different abilities to introduce posttranslational modifications (PTM) on our target allergen, purified proteins were treated with peptide‐N‐glycosidase‐F (PNGase F) for cleavage of N‐linked oligosaccharides. A shift in the SDS‐PAGE migration was noted for M_rFel d 4 after treatment with PNGase F, indicating the removal of N‐glycosylation. After glycoprotein‐specific staining, a significant decrease in signal intensity confirmed deglycosylation of M_rFel d 4 (Figure 3A). The absence of glycosylation in E_rFel d 4 was further confirmed by the removal of the hexa‐histidine tag (~2.2 kDa), which influenced the migration pattern on the SDS‐PAGE, eventually matching the size of deglycosylated M_rFel d 4 (Figure 3A, Figure S1B).

To further determine the accurate molecular masses and investigate the relative abundance of N‐linked glycosylation, we performed high‐resolution mass spectrometry. Analysis of the E_rFel d 4 yielded a mass of 22275.3 Da, in good agreement with the theoretically calculated mass for the hexa‐histidine tagged protein with N‐formylmethionine (22274.9 Da) (Figure S4). In contrast, deconvoluted mass spectra of M_rFel d 4 showed heterogeneity due to the presence of oligosaccharide microheterogeneity. At least 7 glycoforms were detected featuring differences in hexose, N‐acetylhexosamine, fucose, and sialic acid abundance based on the observed mass differences. The mass differences between the theoretically calculated (20035.6 Da) and average observed form (21692.6 Da) suggested the modification of only one glycosylation site (Figure 3B). To verify which of the 2 predicted N‐glycosylation sites carry oligosaccharides, M_rFel d 4 and a portion of the M_rFel d 4 treated with PNGase F were trypsin digested, and the resulting peptides were submitted to LC‐MS/MS analysis. In total, 67% (118/174 residues) of sequence coverage was obtained for M_rFel d 4% and 57% (100/174 residues) for M_rFel d 4 treated with PNGase F (Figure 3C). In untreated M_rFel d 4, we could detect the peptide containing N66 (ALDN^66^SSLSFVFHTK) but not the peptide containing N51. However, after PNGase F deglycosylation of M_rFel d 4, the peptide containing N66 (ALDN^66^SSLSFVFHTK) as well as two peptides containing N51 (IEEN^51^GSMR and EKIEEN^51^GSMR) were detected (Figure 3D,E and Table S3). These results are a clear indirect proof that N51 is N‐glycosylated. Supporting our findings, LC–MS/MS analysis of rFel d 4 produced in *E. coli* (+/− PNGase F treatment) resulted in the detection of both peptides (N51 and N61) independent of PNGase F treatment (Tables S6, S7).

### 3.4 Different IgE Binding Capacities of E_rFel d 4, M_rFel d 4 and M_rFel d 4 PNGase F

Given the presence of N‐glycans in M_rFel d 4, we investigated their potential influence on IgE recognition using ELISA assays where cat‐allergic patient sera were used to determine the IgE‐binding capacity of two rFel d 4 variants. Both proteins exhibited binding profiles to the tested patients' IgE with close mean values of 1.082 and 1.036 for E_rFel d 4 and M_rFel d 4, respectively. However, the individual‐patient analysis showed differences in IgE‐binding capacity of the two rFel d 4 variants (Figure 3F). IgE reactivities of the two variants in patients 1, 3, 6, 8, 9, 12, 15, 16, 18, 19, and 20 were comparable. Patients 7, 11, 13, 14, and 17 showed stronger IgE binding to M_rFel d 4, while patients 4, 5, and 10, reacted strongly with E_rFel d 4 (Table S8). Next, we examined if indeed mammalian glycosylation has an influence on IgE binding to Fel d 4. For this purpose, M_rFel d 4 was deglycosylated by PNGase F treatment, and IgE reactivity of E_rFel d 4, M_rFel d 4, and PNGase F‐deglycosylated Fel d 4 (M_rFel d 4 PNGase F) were compared in additional Fel d 4‐sensitized patients by titration (Tables S9, S10). Results obtained showed that there are sera with stronger IgE reactivity to E_rFel d 4 than to M_rFel d 4 and M_rFel d 4 PNGase F (Tables S9, S10: patients B, C, D, G, H, K, M, O, P). Sera were found which reacted stronger to M_rFel d 4 than to E_rFel d 4 and/or M_rFel d 4 PNGase F (Tables S9, S10: patients A, F, L, R) and there were three sera (Tables S9, S10: patients E, S, T) which reacted stronger to M_rFel d 4 PNGase F than to M_rFel d 4.

**FIGURE 4:**
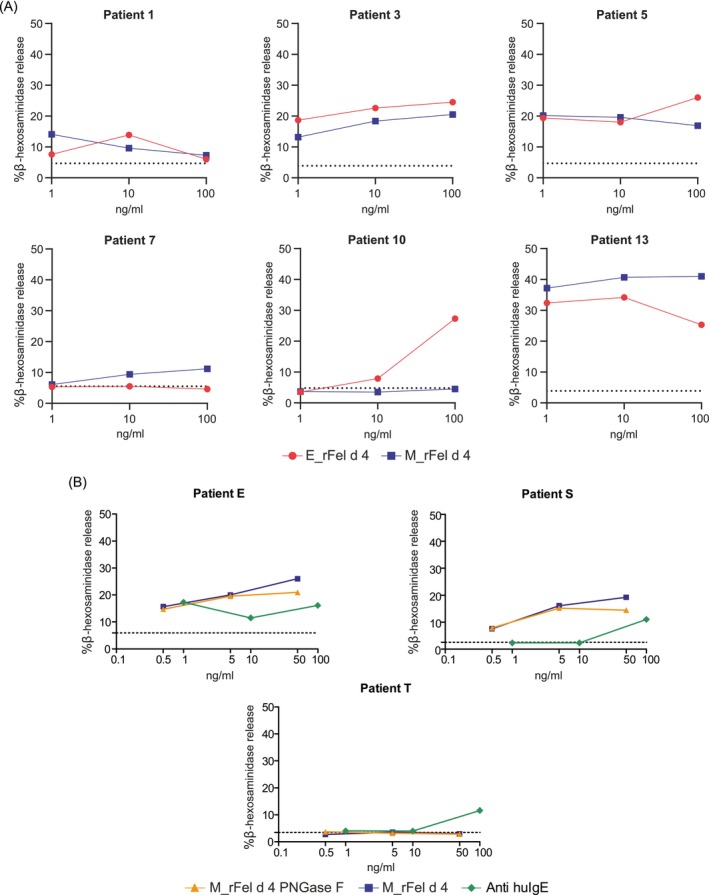
Allergenic activity of the E_rFel d 4 and M_rFel d 4 determined by basophil activation assay. Comparison of the allergenic activity of different concentrations (x‐axis: Concentrations of antigens in ng/ml) of E_rFel d 4 and M_rFel d 4 determined by RBL assay using sera from cat‐allergic patients (patients 1, 3, 5, 7, 10, 13) (A). Results for three additional patients E, S and T tested with M_rFel d 4, M_rFel d 4 PNGase F and anti‐IgE (B). β‐hexosaminidase releases (y‐axes) are expressed as percentages of total mediator contents of cells. The horizontal dashed lines show the percentages of releases observed with patients' sera alone (negative controls).

To assess differences in allergenic activity, we analyzed the potential of the allergens to induce basophil release (Figure 4A, Table S11). Patients 5 and 10 showed a higher release of mediators at the highest concentration of E_rFel d 4 as compared to M_rFel d 4. Patient 13 showed stronger degranulation toward M_rFel d 4, while degranulation was lower for E_rFel d 4. We also performed basophil degranulation experiments for the three sera that had shown stronger IgE reactivity to M_rFel d 4 PNGase F than to M_rFel d 4 (Tables S9, S10: E, S, T) and included an anti‐human IgE antibody for control purposes (Table S12). Both M_rFel d 4 PNGase F and M_rFel d 4 induced comparable basophil activation, reflecting levels of allergen‐specific IgE (Tables S9, S10). Anti‐IgE induced basophil activation (Figure 4B).

### 3.5 Glycosylation Occurs Close to the Major IgE Epitope‐Containing Region

**FIGURE 5:**
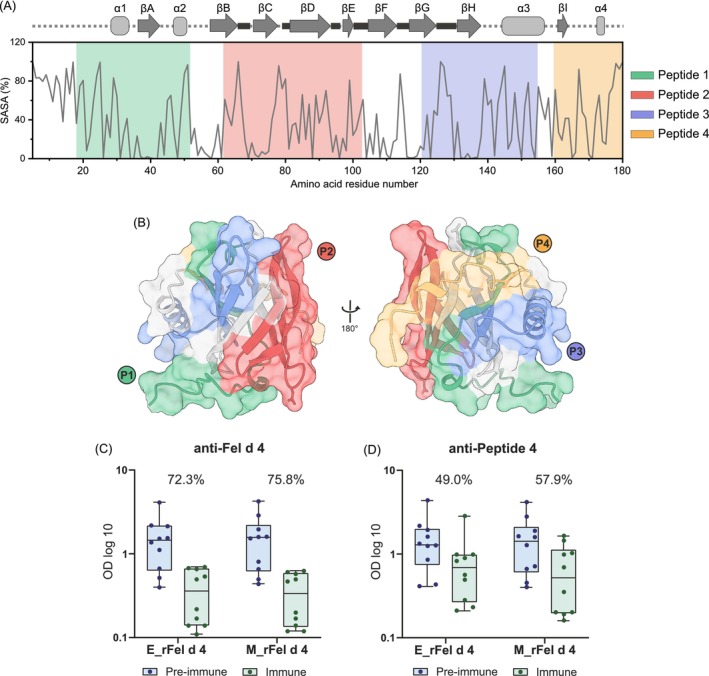
Surface accessibility plot of E_rFel d 4 and Fel d 4‐derived peptides. (A) Solvent accessible surface area (SASA) (y‐axis: %) calculated and plotted per amino acid (x‐axis) using E_rFel d 4 structure coordinates and GetArea [50]. Individual amino acid values are given in Table S13. Secondary structure elements are shown above according to the E_rFel d 4 3D structure. Synthesized peptides are colored. Six additional C‐terminal residues, present in peptide 4, are not included in the SASA plot as they are not modeled in the 3D structure based on which the plot was prepared. The amino acid sequence of Fel d 4‐derived peptides is presented in Table S14. (B) Synthesized peptides displayed on the E_rFel d 4 structure in two orientations, front and back. N‐terminal residues linking the hexa‐histidine tag were excluded for simplicity. (C) Comparison of allergic patients IgE binding (x‐axis: Box‐and‐whisker plots showing minimum, quartiles, median, and maximum values) to E_rFel d 4 or M_rFel d 4 allergen (y‐axis: ODlog10) after preincubation of allergens with rabbit antisera specific for Fel d 4 (green) or for (D) Fel d 4‐derived peptide 4 (green) or corresponding preimmune sera (blue) (C, D) in competitive ELISA. Dots show the individual average OD values (405/492 nm) corresponding to IgE for each of the 10 cat‐allergic patients tested. Mean percentages of inhibition of IgE binding are indicated.

Solvent accessible surface areas (SASA) in Fel d 4 were calculated for individual amino acid side chains (probe particle radius of 1.4 Å) and plotted per residue to uncover regions of high solvent exposure (Figure 5A, Table S13). Antisera specific for one of the four Fel d 4‐derived peptides (Figure 5A,B, Table S14) were tested for their ability to compete with allergic patient IgE binding to Fel d 4. Mapping the surface area occupied by P4 onto the E_rFel d 4 structure, we observed that P4 spans a large part of the back side of the protein surface (Figure 5B). The six C‐terminal residues of P4 were not modeled in the 3D structure due to missing electron density. Notably, P4 is close to the N51‐glycosylation position.

Rabbit Fel d 4‐specific and P4‐specific antibodies were used to evaluate their ability to inhibit the binding of patients' IgE to the recombinant allergens, E_rFel d 4 and M_rFel d 4. Fel d 4‐specific IgG antibodies on average blocked the binding of the patients' IgE to E_rFel d 4 and M_rFel d 4 up to 72.3% and 75.8%, respectively (Figure 5C, Table S15). Importantly, when P4‐specific antisera were used, high inhibition rates were achieved for both recombinant allergen variants (i.e., up to 57.9%) with slightly higher inhibition for M_rFel d 4 than for E_rFel d 4 (Figure 5D, Table S16).

### 3.6 Peptide 4‐Specific IgG Antibodies Can Block the Binding of Patients IgE to rEqu c 1 Allergen and Cross‐React With rEqu c 1 and rCan f 6

**FIGURE 6:**
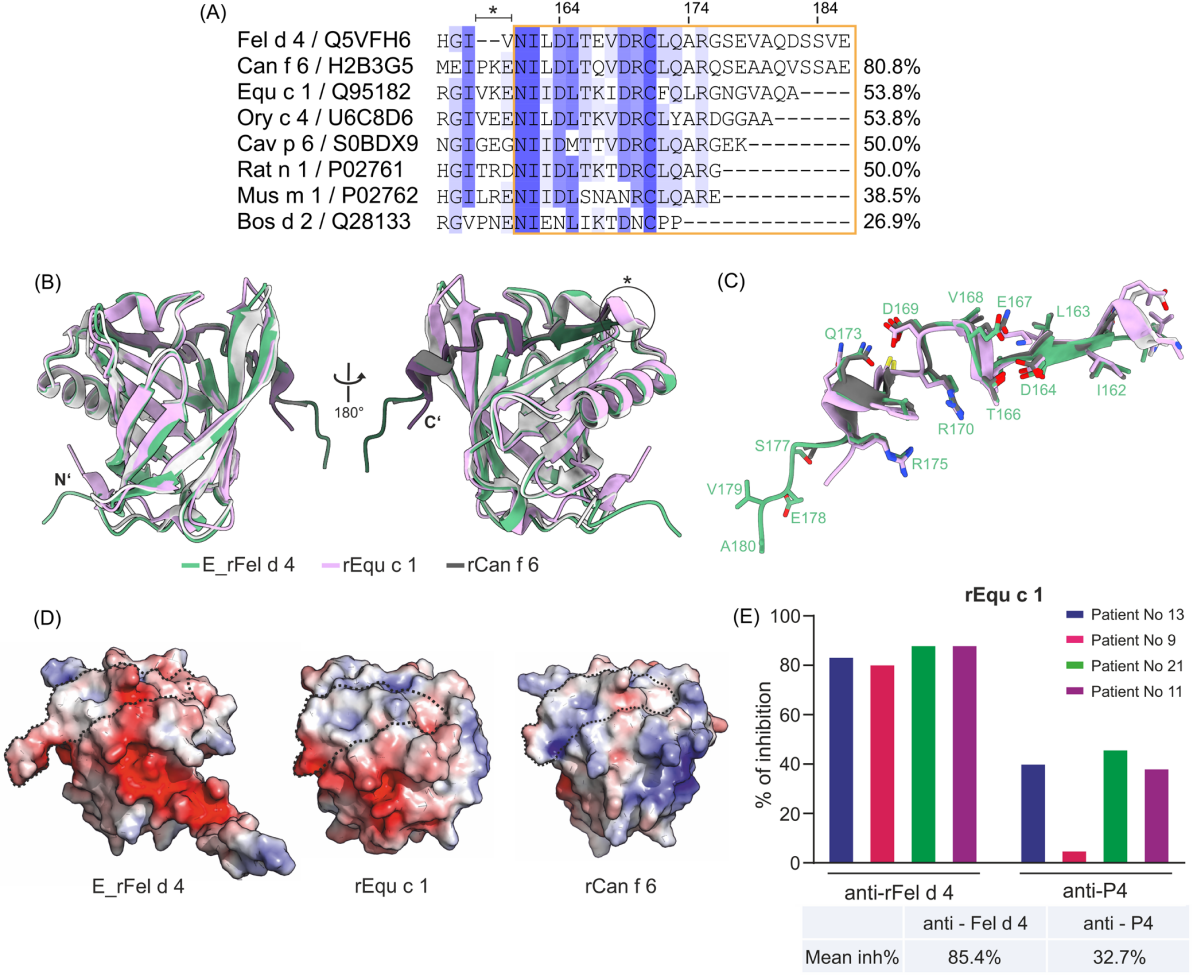
Structural alignment of cross‐reactive allergens and inhibition of IgE binding. (A) Sequence alignment of the Fel d 4 C‐terminus and homologous lipocalin allergens generated using Clustal Omega [51] and displayed with JalView 2.11.3.2 under standard settings [52]. Residues were colored in blue to white color gradient according to percent identity, with a 20% conservation threshold. Residue numbering corresponds to Fel d 4. Asterisk marks the gap in Fel d 4 sequence. P4 is indicated in orange. The percentages of sequence identity of the Fel d 4 peptide and homologous lipocalin sequences are shown on the right margin. (B) Structure alignment for E_rFel d 4 (PDB: 9I2M) (green), rEqu c 1 (PDB: 1EW3) (lilac), and rCan f 6 (PDB: 6NRE) (gray) in cartoon representation and in two orientations. N‐terminal extension in E_rFel d 4 structure is excluded for simplicity. C‐terminal P4 is highlighted in respective darker colors. (C) Superimposed P4 region of E_rFel d 4 (green), rEqu c 1 (lilac), and rCan f 6 (gray) with residue representation and numbering corresponding to E_rFel d 4. (D) Electrostatic surface potential calculated with APBS: rEqu c 1, E_rFel d 4, and rCan f 6 with C‐terminal P4 region surrounded by black dashed line. The gradient coloring from red (−5 kT/e) to blue (5 kT/e) indicates negative and positive potential, respectively. (E) Percentages of inhibition (y‐axis) of patients' IgE binding to major horse allergen Equ c 1 by anti‐Fel d 4 and anti‐P4 rabbit antisera in four Equ c 1 sensitized patients (x‐axis: No 13, 9, 21, 11). The mean percentage of inhibition is shown below.

The tertiary structure of the Fel d 4 was superimposed with 3D structures of the homologous lipocalin allergens from dog (Can f 6), horse (Equ c 1), rabbit (Ory c 4), guinea pig (Cav p 6), rat (Rat n 1), mouse (Mus m 1) and cow (Bos d 2) (Figure S5, Table S17). The overall structures are closely resembling, although the highest structural similarity was found with Can f 6 (CαRMSD: 0.647 Å). Concerning Fel d 4‐derived P4 the highest sequence identity was found for the corresponding peptides of the dog allergen Can f 6 (i.e., 80.8%), followed by the major horse allergen Equ c 1 which showed a considerably lower percentage of sequence identity (i.e., 53.8%) comparable to other lipocalin allergens, whereas the lowest identity was found with Bos d 2 (i.e., 26.9%) (Figure 6A, Figure S6). Multiple sequence alignment in the P4 region revealed a gap of two residues in the Fel d 4 sequence (Figure 6A). This gap influenced the local fold in both Fel d 4 structures, converting the helical turn present at the C‐terminus of most lipocalin structures into a loop in the Fel d 4 structures (Figure 6B,C). Thus, this surface‐exposed part adjacent to P4 remains uncharged in Fel d 4 but has 2 charged residues in the structures of Can f 6 and Equ c 1. A difference in surface electrostatic charge is evident in the modeled P4 (Figure 6D) where an exposed glutamate residue in Fel d 4 (E167) is exchanged for glutamine in Can f 6 and lysine in Equ c 1 (Figure 6C). The amino acid change influences the charge distribution on the allergen surface and could influence the binding strength to cross‐reactive antibodies. When considering the C terminus length, it is apparent that Equ c 1 is four residues shorter than Fel d 4 and Can f 6 (Figure 6A). We investigated whether the major horse allergen Equ c 1 retains sufficient similarity to Fel d 4 in the P4 region to enable IgE cross‐reactivity.

We first tested sera from Equ c 1‐sensitized cat‐allergic patients to study whether Fel d 4‐specific IgG antibodies can inhibit IgE binding to Equ c 1. We found that a Fel d 4‐specific rabbit antiserum was able to strongly inhibit IgE binding to Equ c 1 (mean inhibition: 85.4%), indicating a high cross‐reactivity between the two allergens. Importantly, a rabbit antiserum specific for P4 was also able to substantially inhibit allergic patients' IgE binding to Equ c 1 in three out of the four patients tested (Figure 6E, Table S18). Moreover, rabbit anti‐P4 antibodies reacted not only with E_rFel d 4 but also with rEqu c 1 and rCan f 6 but not with the unrelated birch pollen allergen, rBet v 1 (Figure S7, Table S20). Preimmune serum from the rabbit immunized with P4 showed no reactivity to any of the tested allergens (Figure S7).

## 4 Discussion

More than 200 million patients suffer from an allergy to furry pets, usually experiencing severe respiratory symptoms such as severe chronic rhinitis and asthma [5, 53]. Among recorded cat allergens, Fel d 4 is a highly important allergen due to its frequent recognition, high allergenic activity, and cross‐reactivity with other lipocalin allergens [12, 13, 18, 19]. Given its clinical significance, Fel d 4‐specific forms of treatment, such as allergen‐specific immunotherapy (AIT) or passive immunization strategies with monoclonal antibodies, are needed.

Since diagnostic and therapeutic approaches often use recombinantly produced allergens [54, 55] it is important to note that many allergens, including lipocalins [19], undergo different PTMs that can modify the allergens' IgE‐reactivity [56, 57, 58]. *Escherichia coli* strains, generally used for recombinant allergen production [59], lack the ability to introduce PTMs, importantly, glycosylation due to the lack of the complex glycosylation pathway. With this in mind, we employed two recombinant protein expression systems, *E. coli* and Expi293F, a mammalian cell line, to produce Fel d 4. Both systems generated monomeric and folded proteins in solution. Two N‐glycosylation sites (N^51^GS and N^66^SS) were predicted based on the primary sequence of the allergen. Using mass spectrometry analysis, we could show that E_rFel d 4 is not glycosylated, whereas one site (N51) was glycosylated in M_rFel d 4. Allergen cross‐reactivity can be found between several members of the lipocalin protein family [60, 61]. Risk of cross‐reactivity exists among cat allergen (Fel d 4), dog allergen (Can f 6), and major horse allergen (Equ c 1) [18, 62]. Interestingly, two glycosylation sites at homologous positions were described for Equ c 1; nevertheless, only one site was proven to be N‐glycosylated [63]. In contrast to Fel d 4 and Equ c 1, three putative sites were outlined in Can f 6 [19], whereas no N‐glycosylation sites were predicted in Cav p 6, indicating that glycosylation is not a unique feature in lipocalin allergens. Furthermore, non‐glycosylated E_rFel d 4, glycosylated M_rFel d 4, and deglycosylated M_rFel d 4 PNGase F did not show a significant difference in terms of mean values of IgE‐binding. However, IgE binding to the three forms of Fel d 4 showed some variation when analyzed on individual patient levels (Tables S8–S10). There were sera exhibiting stronger IgE reactivity to E_rFel d 4 than to M_rFel d 4 and M_rFel d 4 PNGase F. Sera were found that reacted stronger to M_rFel d 4 than to E_rFel d 4 and M_rFel d 4 PNGase F, and there were three sera that reacted stronger to M_rFel d 4 PNGase F than to M_rFel d 4. Importantly, stronger IgE reactivity to E_rFel d 4 was basically not associated with stronger IgE reactivity to M_rFel d 4 PNGase F. These results indicate that sera from Fel d 4‐sensitized patients can contain IgE recognizing preferentially M_rFel d 4, others react preferentially with E_rFel d 4 and there are indeed some that reacted better with M_rFel d 4 PNGase F than with M_rFel d 4. For the latter sera, we may consider that carbohydrates indeed shield IgE epitopes, whereas different IgE recognition of E_rFel d 4 and M_rFel d 4 may be due to preferential recognition of IgE epitopes presented in a different way by E_rFel d 4 versus M_rFel d 4. Eventually, a ligand possibly present in E_rFel d 4 may have contributed to its higher IgE binding capacity as it has been described for nonspecific lipid transfer proteins [47]. The higher IgE reactivity of M_rFel d 4 than M_rFel d 4 PNGase F may be attributed to the stabilizing effect of mammalian carbohydrates [64] rather than IgE binding to the carbohydrates on M_rFel d 4, which are unlike IgE‐reactive alpha‐Gal (galactose‐α‐1,3‐galactose (α‐Gal)) [65]. The differences in IgE binding between E_rFel d 4 and M_rFel d 4 were corroborated in the basophil release assays, whereas the slightly higher IgE recognition of M_rFel d 4 PNGase F over M_rFel d 4 observed in approximately 10% of patients did not result in differences regarding allergenic activity in the patients tested.

Fel d 4 crystal structures were obtained from both recombinantly produced allergens. E_rFel d 4 crystallized in a higher symmetry space group, while containing a 22‐amino‐acid extension with an N‐terminal, TEV cleavable hexa‐histidine tag that did not alter the native fold of the molecule, nor did it affect the surface properties. The three‐dimensional structure of the cat allergen Fel d 4 displays a highly conserved lipocalin architecture, closely matching homologous lipocalin allergens (Figure S5, Table S17). Cross‐reactivity between the major horse allergen Equ c 1 and the cat allergen Fel d 4 has been postulated based on their high sequence homology [66] and inhibition experiments [18].

Using rabbit antibodies specific for Fel d 4, IgE reactivity of patients to the major horse allergen Equ c 1 could be inhibited by an average of 85.4% proving high cross‐reactivity between the two allergens. Despite sharing only 53% sequence identity in the P4 region and Equ c 1 having a shorter C‐terminus (Figure 6A) in comparison to Fel d 4, 32.7% inhibition of allergic patients' IgE binding to Equ c 1 was obtained by P4‐specific rabbit antisera. This implies that the C‐terminal region is involved in establishing an important cross‐reactive IgE‐epitope‐containing region also in Equ c 1. The importance of a C‐terminal region of lipocalins in IgE recognition was recognized in another study [21] where Equ c 1 IgE‐epitopes have been analyzed with the use of specific monoclonal antibodies. The authors have shown that reducing the conserved disulfide bond that attaches the C‐terminus to the rest of the structure abolishes antibody binding. This provides further evidence that the binding of the specific antibody depends on the structural integrity of the C‐terminal region. Sequence identity varies a lot between members of the lipocalin allergen family [62] and might not be the limiting factor for their cross‐reactivity, which could be additionally attributed to residue similarity in surface‐exposed regions, fold, PTM, and oligomerization for the formation of the conformational epitopes [67]. Furthermore, the structure of the lipocalin allergen Bos d 5 in complex with the Fab fragment of the human IgE showed that the C‐terminal portion of the allergen is involved in IgE‐binding (PDB: 2R56) [68]. In fact, we were able to show that rabbit antibodies raised against the Fel d 4 P4 peptide cross‐reacted with Equ c 1 and with Can f 6. Accordingly, it seems that the C‐terminal portions of Fel d 4, Equ c 1, Can f 6, and a lipocalin with almost no sequence homology (Bos d 5) are involved in the binding of patients' IgE. Based on this, one could almost assume that IgE antibodies “like” the C‐terminal ends of lipocalin allergens, eventually by selecting and activating specific B cells that bear suitable cognate B‐cell receptors.

In summary, our study is the first to reveal the three‐dimensional structure of the important respiratory allergen Fel d 4 and an IgE‐epitope‐containing patch included in the majority of the polyclonal IgE‐binding repertoire of sensitized patients. This IgE‐binding region also appears in the cross‐reactive allergens, such as the major horse allergen Equ c 1 and Can f 6 from dogs. These results inform about the major IgE‐binding sites and will help to guide targeted allergen‐specific forms of treatment against Fel d 4 and related lipocalin allergens. In fact, recently, the preclinical characterization of a recombinant cat allergy vaccine was reported, which contained Fel d 4‐derived peptide 4 and induced rabbit IgG antibodies cross‐reacting with Equ c 1 [69].

## Author Contributions

**N.T.:** investigation; methodology; formal analysis; data curation; validation; visualization; writing original draft preparation; writing review and editing. **D.T., Z.L.:** investigation; formal analysis; validation; visualization; writing review and editing. **M.C., L.S., T.S., C.G., N.G., R.K.:** investigation; writing review and editing. **B.G., A.W., T.P.K.:** investigation; formal analysis; validation; writing review and editing. **A.K.:** project administration; funding acquisition; writing review and editing; **R.V.:** conceptualization, methodology, data curation, supervision; project administration; funding acquisition; writing review and editing. **W.K.:** conceptualization; methodology; data curation; supervision; project administration; funding acquisition; writing original draft preparation; writing review and editing.

## Conflicts of Interest

Rudolf Valenta has received research grants from Worg Pharmaceuticals, Hangzhou, China, and HVD Biotech, Vienna, Austria, and serves as a consultant for both companies. The authors with a Russian affiliation declare that they have prepared the article in their “personal capacity” and/or that they are employed at an academic/research institution where research or education is the primary function of the entity. The rest of the authors declare no conflicts of interest.

## Supporting information


**Appendix S1:** Supporting Information.

## Data Availability

All of the structural data have been deposited in the Protein Data Bank (http://www.wwpdb.org) with the following links: 8AMC (M_rFel d 4), 9I2M (E_rFel d 4). Additional data can be made available upon reasonable request.
