# Supplementary material for: Major Cat Allergen Fel d 4: Structure and Identification of a Cross‐Reactive IgE‐Epitope‐Containing Area
Source: Allergy. 2025 Nov 24;81(4):1075–88. doi: 10.1111/all.70146 (PMC13040627; doi:10.1111/all.70146)
Supplement: Supplementary file 1 — Appendix S1: Supporting Information. [file ALL-81-1075-s001.docx]

**Supplementary File**

**Major cat allergen Fel d 4: Structure and identification of a cross-reactive IgE-epitope-containing area**

Nikolina Todorović^1^, Daria Trifonova^2,3,4^, Zicheng Liu^2^, Mirela Curin^2^, Laszlo Schooltink^1^, Theo Sagmeister^1^, Christoph Grininger^1^, Renata Kiss^2^, Nina Gottstein^1^, Bernd Gesslbauer^5^, Andreas Winkler^6,7^, Tea Pavkov-Keller^1,7,8^, Alexander Karaulov^3,4^, Rudolf Valenta^2,3,4,9^, Walter Keller^1,7,8,*^

# Supplementary Methods

## Expression and purification of Fel d 4

Recombinant Fel d 4 allergen was expressed in *E. coli* SHuffle T7 Express *lysY* cells (New England Biolabs, Massachusetts, USA). Cells were grown in LB medium supplemented with kanamycin (50 µg/mL) under shaking at 30 °C. At OD_620_ of ~0.5, cells were shifted to 16°C for induction with 1 mM isopropyl-b-D-thiogalactopyranoside (IPTG) and overnight incubation. After harvesting (8000 rpm, 40 min, 4 °C, JLA 8.1000 rotor, Beckman Coulter Avanti J-26 XP, California, USA), Collected cells were resuspended in lysis buffer (50 mM Tris/HCl, pH 8, 150 mM NaCl, 20 mM imidazole) and disrupted with sonication (4x5 min, 70% intensity, 4°C, Sonopuls HD 2070, Bandelin, Germany). Lysates were clarified using centrifugation (20000 rpm, 45 min, 4°C, JA 25.50 rotor, Beckman Coulter Avanti J-26 XP, California, USA), supernatant filtered (0.45 µm, Carl Roth GmbH + Co. KG, Karlsruhe, Germany) and affinity purified on ÄKTA FPLC system using 5mL HisTrap™ HP column (Cytiva, Massachusetts, United States). Protein was eluted (50 mM Tris/HCl, pH 8, 150 mM NaCl, 250 mM imidazole) and dialyzed overnight at 4°C to reduce the Imidazole concentration. Collected protein was subjected to an additional step of size-exclusion chromatography on a Superdex 200 Increase 10/300 GL column (Cytiva, Massachusetts, United States) with the flow rate of 0.375 mL/min, before being concentrated using 10 kDa cutoff membrane (Amicon Ultra-15; Merck Millipore) and stored in buffer (50mM Hepes, pH 7.4, 150 mM NaCl). The purity of E_rFel d 4 was analyzed using 12 % (w/v) SDS-PAGE.

For transient expression of rFel d 4, Expi293F^TM^ cells were cultured in the Expi293F^TM^ Expression Medium (ThermoFisher Scientific) in a suspension, under the specified growth conditions (8 % CO_2_, 37 °C 120 rpm, Orbi-Shaker CO_2_, Benchmark Scientific, New Jersey, USA). Cells were transfected using the ExpiFectamine™ 293 Transfection Kit (ThermoFisher Scientific) according to manufacturer’s instructions. Seventy-two hours after the transfection, the protein-containing supernatant was separated from cell debris (1200 rpm, 10 min, 4°C, A-4-81 rotor, 5810R Eppendorf, Hamburg, Germany) and filtered (0.45 µm, Carl Roth GmbH + Co. KG). Protein was purified with Ni-affinity chromatography with a 5-ml HisTrap™ HP column (Cytiva, Massachusetts, United States). Eluted fractions were dialyzed overnight at 4°C in a dialysis buffer (50mM Tris/HCl, pH 7.4, 150mM NaCl, 20mM imidazole) with histidine-tagged tobacco etch virus (TEV) protease and reverse Ni-affinity purified, collecting the flow through. Purification was finalized with size-exclusion chromatography on a Superdex 200 Increase 10/300 GL (Cytiva, Massachusetts, United States) in buffer (50mM Hepes, pH 7.4, 150 mM NaCl) and collected fractions concentrated (10 kDa, Amicon Ultra-15; Merck Millipore) and protein stored (50mM Hepes, pH 7.4, 150 mM NaCl). The purity of the E_rFel d 4 and M_rFel d 4 was analyzed using 12% (w/v) SDS-PAGE.

## *E. coli* expression and purification of rEqu c 1 and rCan f 6

The major horse allergen Equ c 1 coding sequence (GenBank: AAC48691) or that of Can f 6 (HE653774.1) were codon optimized for *E. coli* expression (Genscript, Piscataway, NJ) and introduced in the pET-27b vector (Novagen, Maddison, USA) and expressed in *E. coli* BL21 GOLD (DE3) (Agilent Technologies, Santa Clara, CA). Cells carrying plasmid DNA were grown in LB medium until an OD600 of 0.3-0.6 was reached, afterwards *E. coli* was cultured in the presence of 1 mM IPTG to produce recombinant allergens for 4 hours at 37°C. The cells were pelleted by centrifugation (7000 rpm, 15 min, 4 °C, R10A5 rotor, HITACHI High-Speed Refrigerated Centrifuge CR 22N, Hitachi Koki Co., Tokyo, Japan). Recombinant allergens were purified under denaturing conditions via Ni-NTA affinity chromatography (Qiagen, Hilden, Germany). Pooled eluted fractions containing purified allergens were dialyzed stepwise with a final buffer against 50 mM NaH_2_PO_4_, pH 8. The concentration of the recombinant allergen was measured using the Micro BCA Assay Kit (ThermoFisher, Pierce, Waltham, MA, USA).

## Circular dichroism spectroscopy

The CD spectroscopy was made on a JASCO (JASCO Corporation, Tokyo, Japan) J-1500 spectropolarimeter. Measurements were carried out at 20°C using a 1 mm path length quartz cuvette. Samples were prepared with concentrations of 0.11-0.13 mg/mL in a 20 mM Phosphate buffer, pH 8, 150 mM NaF. CD spectra were recorded from 260 to 195 nm with 0.2 nm data pitch, 2 nm bandwidth and a scanning speed of 100 nm/min. Spectra were recorded as an average of 10 scans and baseline-corrected by subtracting the corresponding buffer spectra obtained under identical conditions. All spectra were smoothed using a weighted-means algorithm within Spectra manager suite (JASCO Corporation, Tokyo, Japan). Thermal denaturation was assessed under the same measuring parameters with gradually increasing the temperature over range of 20°C to 95°C with increments of 0.1°C at the wavelength of 216 nm. Data points were fitted using Boltzmann sigmoid function. Individual CD spectra were recorded during temperature ramping at 20°C, 95°C and 20°C. The secondary structure was estimated using the BeStSel server [1].

## Protein crystallization

Crystallization experiments were performed with an Oryx8 robot (Douglas Instruments Limited, Hungerford, UK) with the microbatch setup in Vapor batch plate-gold (Douglas instruments, Hungerford, UK) and vapor diffusion sitting drop in SWISSCI 3 Lens crystallization plate (SWISSCI LTD, High Wycombe, UK). M_rFel d 4 was concentrated (10kDa, Amicon Ultra-15; Merck Millipore) and used for microbatch crystallization setup at RT with 1:1 ratio of protein and reservoir solution (**Table S2**) under 2:1 ratio of paraffin and silicon oil. E_rFel d 4 containing hexa-histidine-tag was concentrated (10kDa, Amicon Ultra-15; Merck Millipore) to 11 mg/mL and used for sitting drop crystallization experiments. Drops (1.5 µL) were pipetted at RT in 1:2 ratio of protein and reservoir solution (**Table S2**). The crystallization plates were incubated at 20 °C and obtained crystals were flash-cooled in liquid nitrogen. Crystal screening and data collection were performed at 100 K at the ID23-2 Beamline [2] of the of the European Synchrotron Radiation Facility (ESRF, Grenoble, France) and at the P11 Beamline [3] of the Petra III Deutsches Elektronen-Synchrotron (DESY, Hamburg, Germany), for M_rFel d 4 and E_rFel d 4 crystals, respectively. Data were processed using XDS [4] program package and scaled and merged using AIMLESS [5] to 2.95 Å resolution for M_rFel d 4 (with *P 2_1_* space group and unit cell parameters of a= 67.29Å, b= 120.57 Å, c= 67.15 Å) and to 1.55 Å resolution for E_rFel d 4 (with *I 2 2 2* space group and unit cell parameters of a=64.67 Å, b=77.91 Å, c=79.15 Å).

## Protein glycosylation analysis

Purified proteins were treated with Peptide-N-Glycosidase F (PNGase F, New England Biolabs, MA, USA) according to the manufacturer’s protocol. Proteins were further separated in a 12% (w/v) SDS-PAGE gel and analyzed with Coomassie blue staining and Pro-Q® Emerald 300 Glycoprotein Gel and Blot Stain Kit (ThermoFisher Scientific, MA, USA) by manufacturer’s instructions. For IgE reactivity and RBL assay the deglycosylated M_rFel d 4 (i.e., M_rFel d 4 PNGase F) was further purified by size-exclusion chromatography on a Superdex 200 Increase 10/300 GL (Cytiva, Massachusetts, United States).

## Comparison of IgE reactivity of E_rFel d 4 and M_rFel d 4

The IgE reactivity towards two rFel d 4 allergens was determined by direct ELISA. Plates were coated with Fel d 4 allergens in concentration 2 μg/ml in bicarbonate buffer overnight at 4°C. Plates were washed three times by PBS including 0.5% Tween 20, and blocked by 1% BSA in PBS-Tween 20 (2.5 h, 37°C). Patient serum was added at a 1:10 dilution in 0.5% BSA in PBS-Tween 20 and incubated overnight at 4°C. Plates were washed five times with PBS-Tween 20, and specific patient's IgE bound to the allergens were detected using HRP labelled goat anti-human IgE antibody (KPL, Gaithersburg, MD, USA) at a dilution of 1:2500 in 0.5% BSA in PBS-Tween 20. Results are expressed as the mean OD value of duplicates for each patient. Buffer instead of serum and the sera of non-allergic subject were used for control purposes.

## Titration of E_rFel d 4, M_rFel d 4 and M_rFel d 4 PNGase F-specific IgE antibodies

A titration of IgE levels specific for the three rFel d 4 allergen variants was performed by ELISA. Plates were coated with the Fel d 4 allergens in a concentration 2 μg/ml in bicarbonate buffer overnight at 4 °C. Plates were washed three times by PBS, 0.5% Tween 20, and blocked by 1% BSA in PBS-Tween 20 (2.5 h, 37 °C). Different dilutions of patients’ sera (1:10, 1:20, 1:40, 1:80, 1:160, 1:320) were prepared in PBS, 0.5 % BSA, 0.5% Tween 20 and incubated overnight at 4 °C. Plates were washed five times with PBS-Tween 20, and specific patient’s IgE bound to the allergens were detected using HRP labelled goat anti human IgE antibody (Invitrogen, Waltham, Massachusetts, USA) at a dilution of 1:2500 in PBS-Tween 20, 0.5% BSA. Results are expressed as the average OD value of duplicates for each patient with deviations of <2%. Negative controls were performed by coating human sera albumin, by using buffer instead of serum, and the sera of non-allergic subject were used for control purposes.

## Rat Basophil Leukemia (RBL) assay to determine the allergenic activity of E_rFel d 4, M_rFel d 4 and M_rFel d 4 PNGase F

Rat Basophil Leukemia (RBL) cells expressing human high-affinity IgE receptor FcϵRI (1.5×10^5/well) were incubated in the plate with the serum of allergic subjects at a 1:10 (Figure 4A) or 1:20 (Figure 4B) final dilution overnight. After washing the plates three times with wash buffer (0.1% BSA, 1:1 H2O with 2x Tyrod’s buffer, Sigma, USA), different dilutions of allergens were added (100 ng/ml, 10 ng/ml, 1 ng/ml or 50 ng/ml, 5 ng/ml, 0.5 ng/ml) and mouse anti-human IgE (MAB<IGE>M-7H8 IgG, Roche, Switzerland) at 100 ng/ml, 10 ng/ml, 1 ng/ml. Basophil activation was analyzed by measuring the released β-hexosaminidase in the supernatant. The measurements were performed in duplicates (with less than 5% deviation) or triplicates (Figure 4B) and the result was expressed as an average percentage of the release of β-hexosaminidase compared to the 100% release after the addition of 1% Triton X-100 [6]. The percentage of β-hexosaminidase release from cells incubated with patient sera alone are shown as a cut-off line.

## Comparison of the ability of anti-Fel d 4 and anti-peptide 4 rabbit antisera to inhibit allergic patients IgE binding to allergens by ELISA

Rabbit antisera raised against *E. coli* expressed Fel d 4 and Fel d 4-derived peptide 4 were obtained as described [7]. Plates for competitive ELISA were coated with allergens in a concentration 1 µg/ml in bicarbonate buffer overnight at 4°C. After washing plates 3 times with PBS consisting 0.5% Tween 20, plates were blocked by 1% BSA in PBS-Tween 20 for 2.5 h at 37°C. Plates were incubated with pre-immune (negative control) or specific antisera at a 1:20 dilution in PBS-Tween 20 containing 0.5% BSA overnight at 4°C. After washing plates 5 times by PBS-Tween 20, plates were incubated with the serum of allergic patients at a dilution of 1:10 in 0.5% BSA in PBS-Tween 20 overnight at 4°C. Plates were washed 5 times with PBS-Tween 20, and specific patient's IgE antibodies bound to the allergens were detected using HRP labeled goat anti-human IgE antibodies (KPL, Gaithersburg, MD, USA) at a dilution of 1:2500 in 0.5% BSA in PBS-Tween 20. The percentage of inhibition was calculated according to the difference in IgE binding after incubation with pre-immune and immune rabbit antisera, according to the following formula: 100%−(ODpost/ODpre) × 100%). ODpost and ODpre are optical density (OD) values of IgE binding after pre-incubation with pre-immune or immune rabbit antisera.

## Cross reactivity of rabbit anti-P4 antibodies with Fel d 4, Equ c 1 and Can f 6

A rabbit antiserum specific for Fel d 4-derived peptide 4 was obtained as described [7]. ELISA plates were coated with allergens (E_rFel d 4, rEqu c 1, rCan f 6 and for control purposes, rBet v 1) in a concentration 1 µg/ml in bicarbonate buffer overnight at 4°C. Plates were washed 3 times with PBS, 0.5% Tween 20, blocked with PBS, 1% BSA, 0.5% Tween 20 for 2.5 h at 37°C. Afterwards plates were incubated with pre-immune or anti-P4 antiserum at a 1:200, 1:2000, 1:4000, 1:10000, 1:40000 dilution made in PBS-Tween 20 containing 0.5% BSA overnight at 4°C. After washing plates 5 times by PBS-Tween 20, bound rabbit antibodies were detected using HRP labeled anti rabbit IgG antibodies (Cytiva, Vienna, Austria) at a dilution of 1:2500 in 0.5% BSA in PBS-Tween 20.

# Supplementary Figures and Tables

##
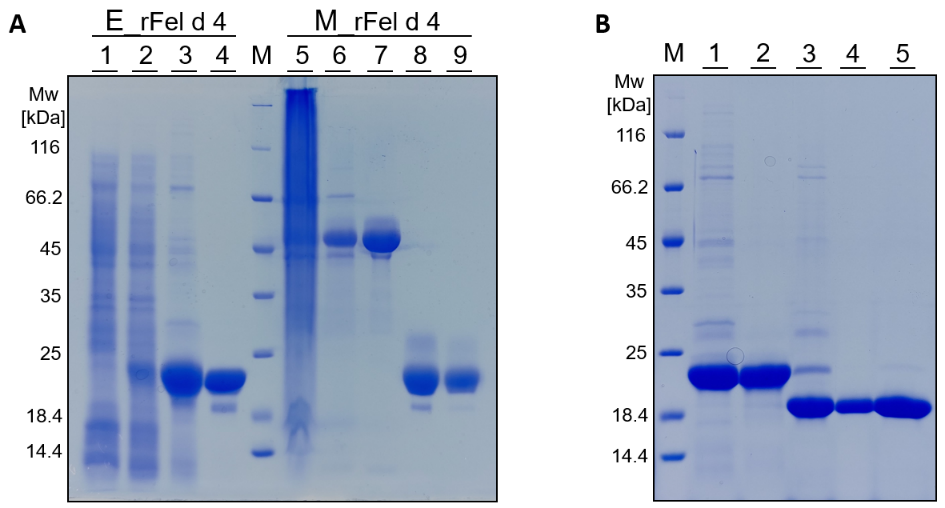
Figure S1. SDS-PAGE monitoring expression and purification of E_rFel d 4 and M_rFel d 4.

(**A**) Coomassie blue stained 12 % (w/v) SDS polyacrylamide gel with E_rFel d 4 and M_rFel d 4 expression and purification. Line 1- *E.coli* uninduced sample (OD_620_-0.6), 2- *E.coli* induced E_rFel d 4 (OD_620_-0.6), 3- Ni^2+^-affinity purified E_rFel d 4, 4- Size-exclusion purified E_rFel d 4, 5- Expi293F cell pellet, 6- Expi293F cell supernatant containing GFP-labeled M_rFel d 4, 7- Ni^2+^-affinity purification of GFP-labeled M_rFel d 4, 8- reverse Ni^2+^-affinity purification of M_rFel d 4 after TEV cleavage of GFP, 9- Size-exclusion chromatography of M_rFel d 4, M- Pierce^TM^ Unstained Protein molecular weight marker (Thermo Fisher Scientific, MA, USA).

(**B**) Coomassie blue stained 12% (w/v) SDS-PAGE monitoring TEV cleavage of E_rFel d 4 and size distribution. Line 1- Ni^2+^-affinity purified E_rFel d 4, 2- Size-exclusion purified E_rFel d 4, 3- TEV cleavage of hexa histidine tag in E_rFel d 4, 4- reverse Ni^2+^-affinity purification of E_rFel d 4 without histidine tag, 5- Size-exclusion chromatography of E_rFel d 4 without histidine tag.

##
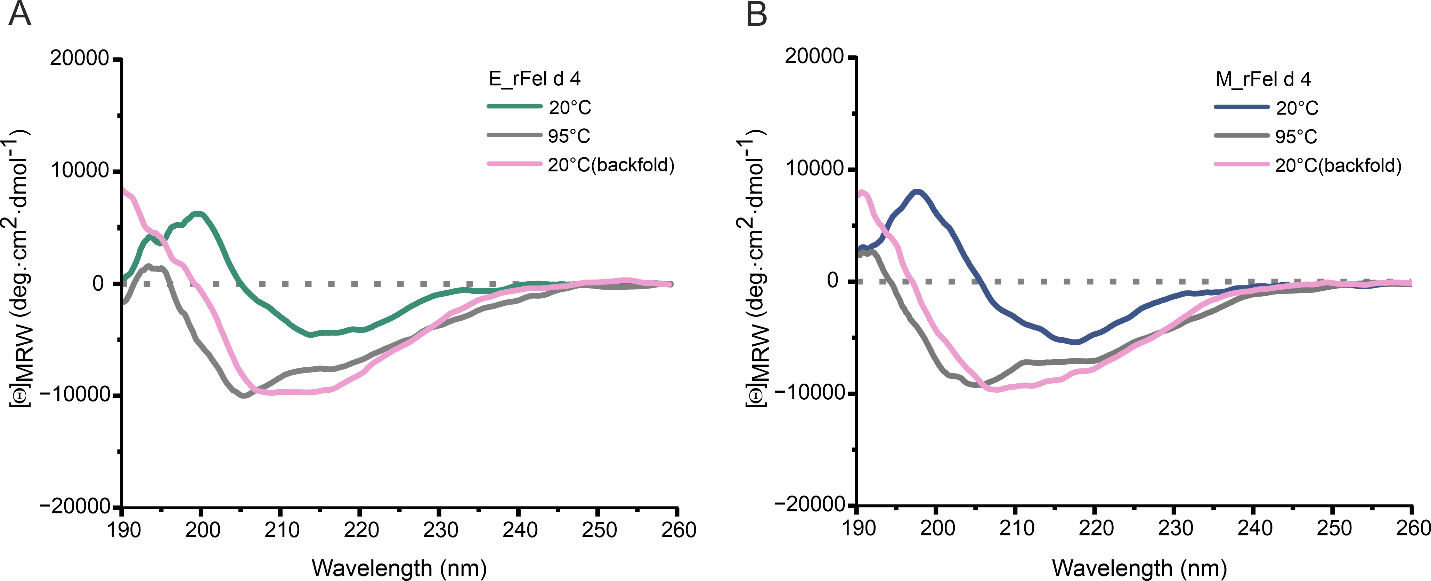
**Figure S2. Circular dichroism spectra during temperature ramping for E_rFel d 4 and M_rFel d 4.**

Temperature induced changes in the circular dichroism spectra recorded at 20 °C before denaturing (green/blue), at 95 °C (gray) and 20 °C after gradual cooling (pink) for E_rFel d 4 (**A**) and M_rFel d 4 (**B**).

## Table S1. Superimposition of E_rFel d 4 structure to 4 monomer chains in asymmetric unit of M_rFel d 4.

| **M_rFel d 4** | **Number of aligned Cα atoms** | **RMSD (Å)** |
| --- | --- | --- |
| Chain A | 153/158 | 0.614 |
| Chain B | 150/158 | 0.622 |
| Chain C | 153/157 | 0.585 |
| Chain D | 154/157 | 0.638 |

Structural alignment of E_rFel d 4 to individual monomer chains A to D of M_rFel d 4. Shown is the number of aligned atoms and RMSD (Å) values.

##
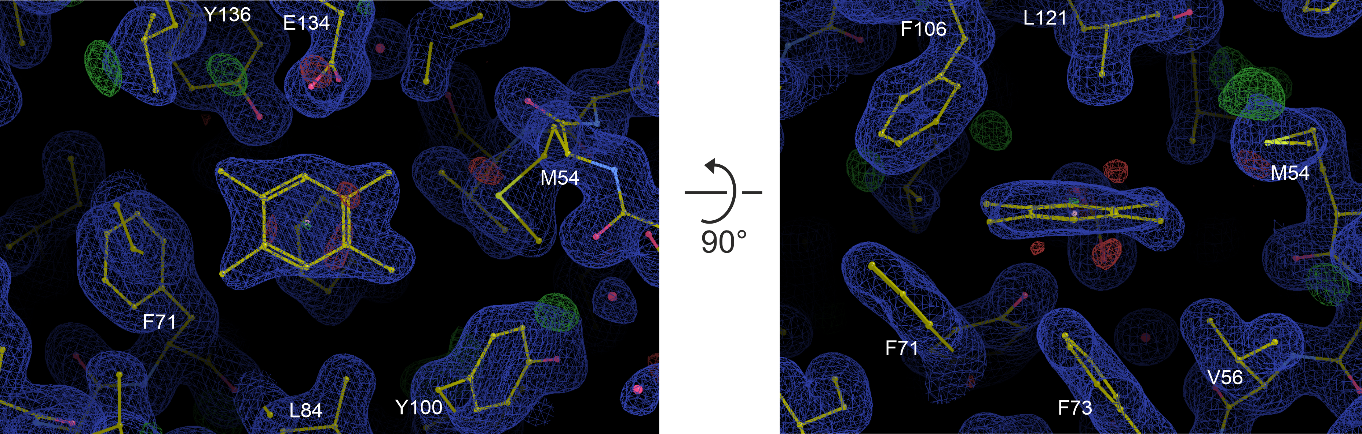
Figure S3. Residual electron density found in the E_rFel d 4 cavity.

2Fo-Fc electron density map (blue mash) of the E_rFel d 4 with the close-up view of the mostly hydrophobic cavity. Residual electron density is observed in the cavity and a Durene molecule (1,2,4,5-tetramethylbenzene) yielded the best fit. The Fo-Fc difference electron density map after refinement is represented in green mash (positive density) and red mash (negative density). Shown are snapshots from Coot with a contour level at 1σ.

## Table S2. Crystallization reservoir composition.

|  | **E_rFel d 4** | **M_rFel d 4** |
| --- | --- | --- |
| **Crystallization condition** | 0.1 M BisTris pH 5.5, 0.2 M NaCl, 25 % (w/v) PEG 3350 | 0.1 M Sodium acetate trihydrate pH 4.5, 2.0 M Ammonium sulfate |

##
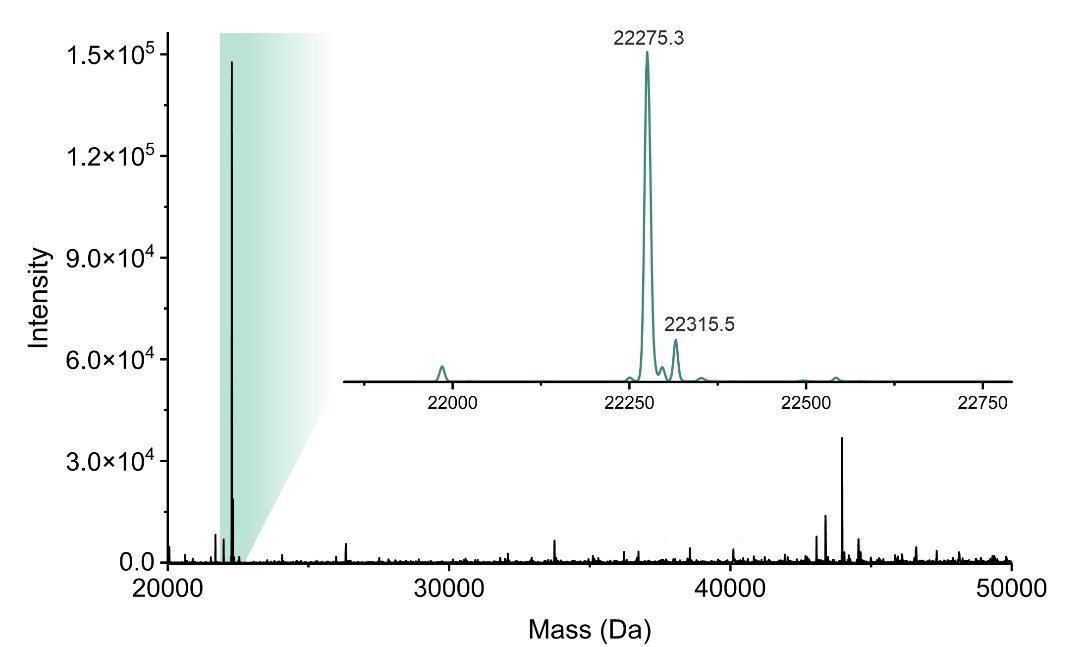
Figure S4. Intact mass analysis and deconvoluted mass spectra of E_rFel d 4.

Deconvoluted mass spectra of E_rFel d 4. The plot of intensity (y-axis) versus mass (x-axis: Da) with inset into region of interest, show the predominant size of E_rFel d 4 corresponding to theoretically calculated mass.

## Table S3. MS/MS identified peptides of M_rFel d 4 and PNGase F treated M_rFel d 4.

| **M_rFel d 4 peptides** | | | | | | | |
| --- | --- | --- | --- | --- | --- | --- | --- |
| **Observed** | **Mr(expt)** | **Mr(calc)** | **Delta** | **Miss** | **Score** | **Expect** | **Peptide** |
| 598.5672 | 1195.1198 | 1194.5955 | 0.5244 | 0 | 85 | 3.4e-009 | K.CTEIFLVADK.T |
| 713.2037 | 1424.3929 | 1423.7381 | 0.6548 | 1 | 36 | 0.00028 | K.CTEIFLVADKTK.D |
| 510.0080 | 1527.0022 | 1525.8041 | 1.1980 | 0 | 67 | 1.9e-007 | K.TRPFQLVEFYAR.E |
| 783.9873 | 1565.9601 | 1564.7886 | 1.1715 | 0 | 74 | 3.6e-008 | K.ALDNSSLSFVFHTK.E |
| 812.7859 | 1623.5573 | 1622.7974 | 0.7600 | 1 | 85 | 2.9e-009 | K.ENGKCTEIFLVADK.T |
| 841.7545 | 1681.4945 | 1679.8770 | 1.6175 | 0 | 106 | 2.8e-011 | K.ISGEWYSILLASDVK.E |
| 927.0582 | 1852.1019 | 1851.9400 | 0.1619 | 2 | 67 | 2.1e-007 | K.ENGKCTEIFLVADKTK.D |
| 970.0167 | 1938.0189 | 1937.0146 | 1.0043 | 1 | 144 | 4e-015 | K.ISGEWYSILLASDVKEK.I |
| 1088.0577 | 2174.1009 | 2173.0474 | 1.0536 | 0 | 125 | 3.4e-013 | K.YCQEHGIVNILDLTEVDR.C |
| 771.1248 | 2310.3526 | 2309.1804 | 1.1722 | 1 | 54 | 3.6e-006 | K.TRPFQLVEFYAREPDVSQK.L |
| 1339.8535 | 4016.5387 | 4014.9819 | 1.5568 | 0 | 52 | 5.7e-006 | K.DGVYTVVYDGYNVFSIVETVYDEYILLHLLNFDK.T |
| 1416.6083 | 4246.8030 | 4244.1246 | 2.6784 | 1 | 45 | 3.2e-005 | K.TKDGVYTVVYDGYNVFSIVETVYDEYILLHLLNFDK.T |
| **M_rFel d 4 PNGase F peptides** | | | | | | | |
| 776.6864 | 775.6791 | 775.4076 | 0.2716 | 0 | 54 | 3.6e-006 | R.SNIDISK.I |
| 468.6881 | 935.3616 | 934.4178 | 0.9438 | 0 | 54 | 4.4e-006 | **K.IEENGSMR.V** |
| 476.1052 | 950.1958 | 950.4127 | -0.2169 | 0 | 47 | 2e-005 | **K.IEENGSMR.V + Oxidation (M)** |
| 597.1495 | 1192.2845 | 1191.5553 | 0.7292 | 1 | 51 | 7.8e-006 | **K.EKIEENGSMR.V** |
| 598.4714 | 1194.9283 | 1194.5955 | 0.3329 | 0 | 88 | 1.4e-009 | K.CTEIFLVADK.T |
| 605.6290 | 1209.2435 | 1207.5503 | 1.6932 | 1 | 45 | 3.2e-005 | **K.EKIEENGSMR.V + Oxidation (M)** |
| 713.0692 | 1424.1239 | 1423.7381 | 0.3858 | 1 | 78 | 1.6e-008 | K.CTEIFLVADKTK.D |
| 510.1036 | 1527.2891 | 1525.8041 | 1.4849 | 0 | 54 | 3.6e-006 | K.TRPFQLVEFYAR.E |
| 784.3498 | 1566.6850 | 1564.7886 | 1.8965 | 0 | 40 | 9.9e-005 | K.ALDNSSLSFVFHTK.E |
| 813.3300 | 1624.6455 | 1622.7974 | 1.8481 | 1 | 46 | 2.3e-005 | K.ENGKCTEIFLVADK.T |
| 841.5753 | 1681.1361 | 1679.8770 | 1.2591 | 0 | 75 | 2.9e-008 | K.ISGEWYSILLASDVK.E |
| 618.8711 | 1853.5915 | 1851.9400 | 1.6514 | 2 | 54 | 4.3e-006 | K.ENGKCTEIFLVADKTK.D |
| 647.5905 | 1939.7495 | 1937.0146 | 2.7350 | 1 | 59 | 1.2e-006 | K.ISGEWYSILLASDVKEK.I |
| 726.0114 | 2175.0122 | 2173.0474 | 1.9649 | 0 | 29 | 0.0012 | K.YCQEHGIVNILDLTEVDR.C |
| 771.6552 | 2311.9436 | 2309.1804 | 2.7632 | 1 | 43 | 5.3e-005 | K.TRPFQLVEFYAREPDVSQK.L |

## Table S4. b and y ion m/z values for peptide IEEN^51^GSMR.

| **#** | **b** | **b^++^** | **b*** | **b*^++^** | **b^0^** | **b^0++^** | **Seq.** | **y** | **y^++^** | **y*** | **y*^++^** | **y^0^** | **y^0++^** | **#** |
| --- | --- | --- | --- | --- | --- | --- | --- | --- | --- | --- | --- | --- | --- | --- |
| 1 | 114.091 | 57.549 |  |  |  |  | **I** |  |  |  |  |  |  | 8 |
| 2 | 243.134 | 122.071 |  |  | 225.123 | 113.065 | **E** | 822.341 | 411.674 | 805.315 | 403.161 | 804.331 | 402.669 | 7 |
| 3 | 372.177 | 186.592 |  |  | 354.166 | 177.587 | **E** | 693.298 | 347.153 | 676.272 | 338.640 | 675.288 | 338.148 | 6 |
| 4 | 486.220 | 243.613 | 469.193 | 235.100 | 468.209 | 234.608 | **N** | 564.256 | 282.632 | 547.229 | 274.118 | 546.245 | 273.626 | 5 |
| 5 | 543.241 | 272.124 | 526.214 | 263.611 | 525.230 | 263.119 | **G** | 450.213 | 225.610 | 433.186 | 217.097 | 432.202 | 216.605 | 4 |
| 6 | 630.273 | 315.640 | 613.246 | 307.127 | 612.262 | 306.635 | **S** | 393.192 | 197.099 | 376.165 | 188.586 | 375.181 | 188.094 | 3 |
| 7 | 761.313 | 381.160 | 744.287 | 372.647 | 743.303 | 372.155 | **M** | 306.159 | 153.583 | 289.133 | 145.070 |  |  | 2 |
| 8 |  |  |  |  |  |  | **R** | 175.119 | 88.063 | 158.092 | 79.550 |  |  | 1 |

## Table S5. b and y ion m/z values for peptide EKIEEN^51^GSMR.

| **#** | **b** | **b^++^** | **b*** | **b*^++^** | **b^0^** | **b^0++^** | **Seq.** | **y** | **y^++^** | **y*** | **y*^++^** | **y^0^** | **y^0++^** | **#** |
| --- | --- | --- | --- | --- | --- | --- | --- | --- | --- | --- | --- | --- | --- | --- |
| 1 | 130.050 | 65.529 |  |  | 112.039 | 56.523 | **E** |  |  |  |  |  |  | 10 |
| 2 | 258.145 | 129.576 | 241.118 | 121.063 | 240.134 | 120.571 | **K** | 1063.520 | 532.264 | 1046.494 | 523.750 | 1045.510 | 523.258 | 9 |
| 3 | 371.229 | 186.118 | 354.202 | 177.605 | 353.218 | 177.113 | **I** | 935.425 | 468.216 | 918.399 | 459.703 | 917.415 | 459.211 | 8 |
| 4 | 500.272 | 250.639 | 483.245 | 242.126 | 482.261 | 241.634 | **E** | 822.341 | 411.674 | 805.315 | 403.161 | 804.331 | 402.669 | 7 |
| 5 | 629.314 | 315.161 | 612.288 | 306.647 | 611.304 | 306.155 | **E** | 693.298 | 347.153 | 676.272 | 338.640 | 675.288 | 338.148 | 6 |
| 6 | 743.357 | 372.182 | 276.331 | 363.669 | 725.346 | 363.177 | **N** | 564.256 | 282.632 | 547.229 | 274.118 | 546.245 | 273.626 | 5 |
| 7 | 800.379 | 400.693 | 783.352 | 392.180 | 782.368 | 391.688 | **G** | 450.213 | 225.610 | 433.186 | 217.097 | 432.202 | 216.605 | 4 |
| 8 | 887.411 | 444.209 | 870.384 | 435.696 | 869.400 | 435.204 | **S** | 393.159 | 197.099 | 376.165 | 188.586 | 375.181 | 188.094 | 3 |
| 9 | 1018.451 | 509.729 | 1001.424 | 501.216 | 1000.440 | 500.724 | **M** | 306.159 | 153.583 | 289.133 | 145.070 |  |  | 2 |
| 10 |  |  |  |  |  |  | **R** | 175.119 | 88.063 | 158.092 | 79.550 |  |  | 1 |

## Table S6. MS/MS identified peptides of E_rFel d 4.

| **E_rFel d 4 peptides** | | | | | | | |
| --- | --- | --- | --- | --- | --- | --- | --- |
| **Observed** | **Mr(expt)** | **Mr(calc)** | **Delta** | **Miss** | **Score** | **Expect** | **Peptide** |
| 776.7153 | 775.7080 | 775.4076 | 0.3004 | 0 | 41 | 8.1e-005 | R.SNIDISK.I |
| 477.1280 | 952.2415 | 950.4127 | 1.8288 | 0 | 26 | 0.0023 | **K.IEENGSMR.V + Oxidation (M)** |
| 597.2819 | 1192.5492 | 1191.5553 | 0.9938 | 1 | 31 | 0.00074 | **K.EKIEENGSMR.V** |
| 605.4730 | 1208.9315 | 1207.5503 | 1.3812 | 1 | 28 | 0.0015 | **K.EKIEENGSMR.V + Oxidation (M)** |
| 598.6566 | 1195.2986 | 1194.5955 | 0.7031 | 0 | 81 | 7.1e-009 | K.CTEIFLVADK.T |
| 713.3358 | 1424.6571 | 1423.7381 | 0.9190 | 1 | 83 | 4.7e-009 | K.CTEIFLVADKTK.D |
| 764.1270 | 1526.2395 | 1525.8041 | 0.4353 | 0 | 63 | 4.6e-007 | K.TRPFQLVEFYAR.E |
| 783.8867 | 1565.7589 | 1564.7886 | 0.9703 | 0 | 76 | 2.4e-008 | K.ALDNSSLSFVFHTK.E |
| 812.5797 | 1623.1449 | 1622.7974 | 0.3475 | 1 | 77 | 2.1e-008 | K.ENGKCTEIFLVADK.T |
| 841.8940 | 1681.7735 | 1679.8770 | 1.8965 | 0 | 108 | 1.5e-011 | K.ISGEWYSILLASDVK.E |
| 618.6681 | 1852.9824 | 1851.9400 | 1.0424 | 2 | 44 | 4.2e-005 | K.ENGKCTEIFLVADKTK.D |
| 970.2227 | 1938.4308 | 1937.0146 | 1.4162 | 1 | 129 | 1.3e-013 | K.ISGEWYSILLASDVKEK.I |
| 1087.6852 | 2173.3558 | 2173.0474 | 0.3084 | 0 | 125 | 3.5e-013 | K.YCQEHGIVNILDLTEVDR.C |
| 771.0934 | 2310.2583 | 2309.1804 | 1.0779 | 1 | 52 | 6.9e-006 | K.TRPFQLVEFYAREPDVSQK.L |
| 1139.6035 | 3415.7887 | 3414.4834 | 1.3053 | 0 | 79 | 1.3e-008 | M.GSSHHHHHHSSGENLYFQGHMHEEENVVR.S |
| 1145.0657 | 3432.1752 | 3430.4783 | 1.6969 | 0 | 83 | 4.8e-009 | M.GSSHHHHHHSSGENLYFQGHMHEEENVVR.S + Oxidation(M) |

## Table S7. MS/MS identified peptides of PNGase F treated E_rFel d 4.

| **PNGase treated E_rFel d 4 peptides** | | | | | | | |
| --- | --- | --- | --- | --- | --- | --- | --- |
| **Observed** | **Mr(expt)** | **Mr(calc)** | **Delta** | **Miss** | **Score** | **Expect** | **Peptide** |
| 776.6864 | 775.6791 | 775.4076 | 0.2716 | 0 | 54 | 3.6e-006 | R.SNIDISK.I |
| 468.6881 | 935.3616 | 934.4178 | 0.9438 | 0 | 54 | 4.4e-006 | **K.IEENGSMR.V** |
| 476.1052 | 950.1958 | 950.4127 | -0.2169 | 0 | 47 | 2e-005 | **K.IEENGSMR.V + Oxidation (M)** |
| 597.1495 | 1192.2845 | 1191.5553 | 0.7292 | 1 | 51 | 7.8e-006 | **K.EKIEENGSMR.V** |
| 605.6290 | 1209.2435 | 1207.5503 | 1.6932 | 1 | 45 | 3.2e-005 | **K.EKIEENGSMR.V + Oxidation (M)** |
| 598.4714 | 1194.9283 | 1194.5955 | 0.3329 | 0 | 88 | 1.4e-009 | K.CTEIFLVADK.T |
| 713.0692 | 1424.1239 | 1423.7381 | 0.3858 | 1 | 78 | 1.6e-008 | K.CTEIFLVADKTK.D |
| 510.1036 | 1527.2891 | 1525.8041 | 1.4849 | 0 | 54 | 3.6e-006 | K.TRPFQLVEFYAR.E |
| 784.3498 | 1566.6850 | 1564.7886 | 1.8965 | 0 | 40 | 9.9e-005 | K.ALDNSSLSFVFHTK.E |
| 813.3300 | 1624.6455 | 1622.7974 | 1.8481 | 1 | 46 | 2.3e-005 | K.ENGKCTEIFLVADK.T |
| 841.5753 | 1681.1361 | 1679.8770 | 1.2591 | 0 | 75 | 2.9e-008 | K.ISGEWYSILLASDVK.E |
| 618.8711 | 1853.5915 | 1851.9400 | 1.6514 | 2 | 54 | 4.3e-006 | K.ENGKCTEIFLVADKTK.D |
| 647.5905 | 1939.7495 | 1937.0146 | 2.7350 | 1 | 59 | 1.2e-006 | K.ISGEWYSILLASDVKEK.I |
| 726.0114 | 2175.0122 | 2173.0474 | 1.9649 | 0 | 29 | 0.0012 | K.YCQEHGIVNILDLTEVDR.C |
| 771.6552 | 2311.9436 | 2309.1804 | 2.7632 | 1 | 43 | 5.3e-005 | K.TRPFQLVEFYAREPDVSQK.L |
| 1145.2327 | 3432.6762 | 3430.4783 | 2.1979 | 0 | 16 | 0.0024 | M.GSSHHHHHHSSGENLYFQGHMHEEENVVR.S + Oxidation (M) |

## Table S8. Comparison of the E_rFel d 4 and M_rFel d 4 for IgE reactivity by ELISA.

|  | **OD (rFel d 4)** | |
| --- | --- | --- |
| **Patient** | **E_rFel d 4** | **M_rFel d 4** |
| P1 | 0.491 | 0.486 |
| P3 | 1.755 | 1.731 |
| **P4** | **1.957** | **1.116** |
| **P5** | **1.044** | **0.877** |
| P6 | 2.241 | 2.303 |
| **P7** | **0.317** | **0.425** |
| P8 | 0.155 | 0.149 |
| P9 | 0.469 | 0.515 |
| **P10** | **3.424** | **2.006** |
| **P11** | **1.768** | **2.147** |
| P12 | 0.407 | 0.471 |
| **P13** | **1.322** | **1.672** |
| **P14** | **3.437** | **3.837** |
| P15 | 0.336 | 0.326 |
| P16 | 0.328 | 0.349 |
| **P17** | **0.210** | **0.305** |
| P18 | 0.307 | 0.398 |
| P19 | 0.333 | 0.306 |
| P20 | 0.265 | 0.268 |
| **Mean** | **1.082** | **1.036** |
| Buf. ctrl | 0.094 |  |

Average OD values corresponding to the bound patients´ IgE to Fel d 4 allergens. Results expressed as means of duplicates with a deviation < 5%. Values were colored according to IgE binding preference. Namely, patients showing higher IgE binding towards E_rFel d 4 are presented in green and the ones showing higher IgE reactivity towards M_rFel d 4 are in blue.

## Table S9. IgE reactivity titration for E_rFel d 4 and M_rFel d 4.

|  | **E_rFel d 4** | | | | | |  | **M_rFel d 4** | | | | | |
| --- | --- | --- | --- | --- | --- | --- | --- | --- | --- | --- | --- | --- | --- |
| **Patient** | **1:10** | **1:20** | **1:40** | **1:80** | **1:160** | **1:320** |  | **1:10** | **1:20** | **1:40** | **1:80** | **1:160** | **1:320** |
| **A** | 0.328 | 0.212 | 0.188 | 0.153 | 0.132 | 0.141 |  | 0.374 | 0.248 | 0.194 | 0.193 | 0.198 | 0.197 |
| **B** | 0.389 | 0.232 | 0.185 | 0.139 | 0.110 | 0.109 |  | 0.346 | 0.219 | 0.154 | 0.115 | 0.100 | 0.104 |
| **C** | 0.976 | 0.544 | 0.342 | 0.233 | 0.167 | 0.150 |  | 0.957 | 0.541 | 0.295 | 0.196 | 0.149 | 0.132 |
| **D** | 0.260 | 0.186 | 0.153 | 0.130 | 0.112 | 0.109 |  | 0.238 | 0.170 | 0.129 | 0.109 | 0.098 | 0.097 |
| **E** | 0.461 | 0.291 | 0.203 | 0.170 | 0.141 | 0.129 |  | 0.567 | 0.296 | 0.242 | 0.153 | 0.135 | 0.137 |
| **F** | 1.128 | 0.643 | 0.414 | 0.253 | 0.182 | 0.148 |  | 1.307 | 0.647 | 0.406 | 0.233 | 0.161 | 0.133 |
| **G** | 0.352 | 0.236 | 0.175 | 0.139 | 0.133 | 0.128 |  | 0.321 | 0.224 | 0.157 | 0.127 | 0.126 | 0.132 |
| **H** | 0.164 | 0.124 | 0.130 | 0.123 | 0.098 | 0.102 |  | 0.132 | 0.113 | 0.113 | 0.109 | 0.086 | 0.088 |
| **I** | 0.318 | 0.206 | 0.168 | 0.139 | 0.123 | 0.139 |  | 0.307 | 0.200 | 0.155 | 0.124 | 0.108 | 0.113 |
| **J** | 0.668 | 0.398 | 0.263 | 0.193 | 0.151 | 0.136 |  | 0.676 | 0.391 | 0.254 | 0.180 | 0.144 | 0.124 |
| **K** | 0.898 | 0.517 | 0.331 | 0.230 | 0.178 | 0.152 |  | 0.865 | 0.502 | 0.303 | 0.217 | 0.160 | 0.140 |
| **L** | 0.185 | 0.152 | 0.127 | 0.113 | 0.107 | 0.105 |  | 0.204 | 0.149 | 0.114 | 0.098 | 0.092 | 0.090 |
| **M** | 0.199 | 0.168 | 0.134 | 0.119 | 0.111 | 0.116 |  | 0.172 | 0.138 | 0.122 | 0.111 | 0.106 | 0.115 |
| **N** | 0.224 | 0.179 | 0.154 | 0.143 | 0.136 | 0.126 |  | 0.236 | 0.167 | 0.140 | 0.128 | 0.125 | 0.118 |
| **O** | 0.222 | 0.155 | 0.130 | 0.114 | 0.106 | 0.110 |  | 0.202 | 0.139 | 0.113 | 0.100 | 0.098 | 0.100 |
| **P** | 0.289 | 0.178 | 0.132 | 0.109 | 0.100 | 0.098 |  | 0.262 | 0.176 | 0.123 | 0.104 | 0.100 | 0.094 |
| **Q** | 0.504 | 0.304 | 0.211 | 0.153 | 0.130 | 0.117 |  | 0.500 | 0.294 | 0.198 | 0.146 | 0.118 | 0.109 |
| **R** | 0.375 | 0.315 | 0.230 | 0.185 | 0.166 | 0.159 |  | 0.533 | 0.468 | 0.332 | 0.238 | 0.194 | 0.161 |
| **S** | 0.363 | 0.296 | 0.262 | 0.198 | 0.155 | 0.150 |  | 0.724 | 0.344 | 0.275 | 0.186 | 0.154 | 0.136 |
| **T** | 0.258 | 0.221 | 0.146 | 0.103 | 0.124 | 0.143 |  | 0.155 | 0.171 | 0.135 | 0.100 | 0.094 | 0.131 |
| **N1** | 0.138 | 0.127 | 0.128 | 0.131 | 0.128 | 0.136 |  | 0.120 | 0.115 | 0.106 | 0.112 | 0.115 | 0.134 |
| **N2** | 0.152 | 0.141 | 0.145 | 0.139 | 0.144 | 0.143 |  | 0.134 | 0.112 | 0.116 | 0.109 | 0.114 | 0.133 |
| **N3** | 0.130 | 0.115 | 0.110 | 0.109 | 0.108 | 0.111 |  | 0.126 | 0.105 | 0.101 | 0.095 | 0.096 | 0.102 |
| **N4** | 0.115 | 0.107 | 0.104 | 0.097 | 0.100 | 0.101 |  | 0.111 | 0.103 | 0.092 | 0.091 | 0.096 | 0.099 |

*Abbreviation: N- non-allergic

## Table S10. IgE reactivity titration for M_rFel d 4 PNGase F and HSA.

|  | **M_rFel d 4 PNGase F** | | | | | |  | **HSA** | | | | | |
| --- | --- | --- | --- | --- | --- | --- | --- | --- | --- | --- | --- | --- | --- |
| **Patient** | **1:10** | **1:20** | **1:40** | **1:80** | **1:160** | **1:320** |  | **1:10** | **1:20** | **1:40** | **1:80** | **1:160** | **1:320** |
| **A** | 0.267 | 0.247 | 0.239 | 0.175 | 0.165 | 0.216 |  | 0.226 | 0.250 | 0.297 | 0.184 | 0.152 | 0.231 |
| **B** | 0.240 | 0.173 | 0.130 | 0.104 | 0.096 | 0.100 |  | 0.115 | 0.092 | 0.098 | 0.088 | 0.086 | 0.098 |
| **C** | 0.553 | 0.321 | 0.207 | 0.149 | 0.122 | 0.120 |  | 0.115 | 0.105 | 0.111 | 0.098 | 0.099 | 0.107 |
| **D** | 0.204 | 0.148 | 0.115 | 0.099 | 0.096 | 0.097 |  | 0.087 | 0.085 | 0.079 | 0.081 | 0.079 | 0.090 |
| **E** | 0.641 | 0.347 | 0.209 | 0.152 | 0.131 | 0.136 |  | 0.110 | 0.110 | 0.112 | 0.113 | 0.106 | 0.129 |
| **F** | 1.082 | 0.589 | 0.383 | 0.222 | 0.152 | 0.130 |  | 0.110 | 0.102 | 0.155 | 0.105 | 0.096 | 0.116 |
| **G** | 0.201 | 0.165 | 0.125 | 0.113 | 0.110 | 0.116 |  | 0.110 | 0.098 | 0.098 | 0.094 | 0.100 | 0.113 |
| **H** | 0.132 | 0.120 | 0.117 | 0.105 | 0.085 | 0.088 |  | 0.123 | 0.102 | 0.102 | 0.102 | 0.083 | 0.088 |
| **I** | 0.272 | 0.174 | 0.139 | 0.116 | 0.107 | 0.109 |  | 0.110 | 0.090 | 0.088 | 0.088 | 0.094 | 0.105 |
| **J** | 0.559 | 0.336 | 0.214 | 0.159 | 0.139 | 0.122 |  | 0.105 | 0.095 | 0.098 | 0.100 | 0.099 | 0.102 |
| **K** | 0.799 | 0.479 | 0.282 | 0.213 | 0.155 | 0.137 |  | 0.150 | 0.145 | 0.134 | 0.132 | 0.123 | 0.121 |
| **L** | 0.187 | 0.146 | 0.116 | 0.098 | 0.092 | 0.092 |  | 0.089 | 0.091 | 0.089 | 0.087 | 0.088 | 0.090 |
| **M** | 0.146 | 0.134 | 0.122 | 0.112 | 0.107 | 0.108 |  | 0.124 | 0.120 | 0.107 | 0.107 | 0.097 | 0.107 |
| **N** | 0.239 | 0.159 | 0.136 | 0.127 | 0.124 | 0.118 |  | 0.122 | 0.109 | 0.112 | 0.110 | 0.114 | 0.116 |
| **O** | 0.178 | 0.123 | 0.107 | 0.097 | 0.096 | 0.101 |  | 0.134 | 0.089 | 0.088 | 0.090 | 0.088 | 0.094 |
| **P** | 0.231 | 0.163 | 0.116 | 0.105 | 0.097 | 0.101 |  | 0.096 | 0.083 | 0.078 | 0.083 | 0.086 | 0.090 |
| **Q** | 0.424 | 0.274 | 0.183 | 0.135 | 0.115 | 0.109 |  | 0.112 | 0.097 | 0.093 | 0.088 | 0.091 | 0.103 |
| **R** | 0.541 | 0.412 | 0.295 | 0.237 | 0.200 | 0.164 |  | 0.127 | 0.137 | 0.141 | 0.140 | 0.152 | 0.126 |
| **S** | 0.806 | 0.384 | 0.267 | 0.181 | 0.148 | 0.161 |  | 0.219 | 0.098 | 0.093 | 0.098 | 0.106 | 0.129 |
| **T** | 0.164 | 0.177 | 0.103 | 0.103 | 0.080 | 0.119 |  | 0.142 | 0.157 | 0.122 | 0.090 | 0.115 | 0.156 |
| **N1** | 0.121 | 0.112 | 0.103 | 0.109 | 0.120 | 0.144 |  | 0.116 | 0.101 | 0.098 | 0.108 | 0.107 | 0.132 |
| **N2** | 0.150 | 0.110 | 0.107 | 0.112 | 0.113 | 0.132 |  | 0.129 | 0.108 | 0.106 | 0.106 | 0.112 | 0.128 |
| **N3** | 0.142 | 0.100 | 0.101 | 0.099 | 0.097 | 0.104 |  | 0.110 | 0.095 | 0.091 | 0.089 | 0.092 | 0.101 |
| **N4** | 0.112 | 0.110 | 0.092 | 0.093 | 0.090 | 0.096 |  | 0.127 | 0.114 | 0.091 | 0.087 | 0.088 | 0.100 |

*Abbreviation: HSA- human serum albumin; N- non-allergic

**Table S11. Percentage of β- hexosaminidase release for E_rFel d 4 and M_rFel d 4.**

|  | **100 ng/ml** | | **10 ng/ml** | | **1 ng/ml** | | **IgE (OD)** | |
| --- | --- | --- | --- | --- | --- | --- | --- | --- |
| **Patient** | **E_rFel d 4** | **M_rFel d 4** | **E_rFel d 4** | **M_rFel d 4** | **E_rFel d 4** | **M_rFel d 4** | **E_rFel d 4** | **M_rFel d 4** |
| P1 | 6.0 | 7.3 | 13.9 | 9.6 | 7.6 | 14.1 | 0.49 | 0.49 |
| P3 | 24.5 | 20.5 | 22.6 | 18.4 | 18.7 | 13.2 | 1.75 | 1.73 |
| P5 | 26.0 | 16.9 | 18.0 | 19.6 | 19.4 | 20.2 | 1.04 | 0.88 |
| P7 | 4.6 | 11.2 | 5.5 | 9.4 | 5.3 | 6.1 | 0.32 | 0.42 |
| P10 | 27.3 | 4.5 | 7.9 | 3.5 | 3.5 | 3.7 | 3.62 | 2.01 |
| P13 | 25.3 | 41.0 | 34.2 | 40.7 | 32.4 | 37.2 | 1.32 | 1.67 |
| P17 | 4.7 | 4.1 | 6.7 | 5.4 | 5.7 | 6.6 | 0.21 | 0.30 |

Shown are percent averages of duplicate measurements of β-hexosaminidase release for different concentrations of E_rFel d 4 and M_rFel d 4 using different patient serum.

## Table S12. Percentage of β- hexosaminidase release for M_rFel d 4, M_rFel d 4 PNGase F and anti-human IgE antibody.

| **Patient** | **M_rFel d 4** | | | **M_rFel d 4 PNGase F** | | | **Anti-IgE** | | | |
| --- | --- | --- | --- | --- | --- | --- | --- | --- | --- | --- |
|  | **0.5 ng/ml** | **5 ng/ml** | **50 ng/ml** | **0.5 ng/ml** | **5 ng/ml** | **50 ng/ml** | **1 ng/ml** | **10 ng/ml** | **100 ng/ml** |  |
| E | 15.67 | 20.03 | 26.03 | 14.74 | 19.58 | 20.96 | 17.25 | 11.54 | 16.14 |  |
| S | 7.58 | 16.10 | 19.22 | 7.87 | 15.24 | 14.48 | 2.33 | 2.30 | 11.04 |  |
| T | 2.68 | 3.58 | 2.92 | 3.68 | 3.16 | 2.82 | 4.04 | 3.97 | 11.60 |  |

Shown are percent averages of triplicate measurements of β-hexosaminidase release for different concentrations of M_rFel d 4, M_rFel d 4 PNGase F and anti-human IgE antibody (MAB<IGE>M-7H8 IgG, Roche, Switzerland) using different patient serum.

**Table S13. SASA (%) values per amino acid.**

| **Amino acid** | **SASA (%)** | **Amino acid** | **SASA (%)** | **Amino acid** | **SASA (%)** | **Amino acid** | **SASA (%)** | **Amino acid** | **SASA (%)** | **Amino acid** | **SASA (%)** | **Amino acid** | **SASA (%)** |
| --- | --- | --- | --- | --- | --- | --- | --- | --- | --- | --- | --- | --- | --- |
| **Ser5** | 100 | **Ile31** | 0.5 | **Phe57** | 3 | **Glu83** | 36.7 | **Val109** | 21.5 | **Phe135** | 0 | **Asn161** | 26.8 |
| **Gly6** | 83.1 | **Ser32** | 25.3 | **Val58** | 0 | **Ile84** | 15.8 | **Glu110** | 4.4 | **Tyr136** | 0.8 | **Ile162** | 18.9 |
| **Glu7** | 83.5 | **Gly33** | 41.4 | **Glu59** | 13.2 | **Phe85** | 66 | **Thr111** | 0 | **Ala137** | 1.7 | **Leu163** | 41.6 |
| **Asn8** | 79.6 | **Glu34** | 0 | **His60** | 35.1 | **Leu86** | 19.7 | **Val112** | 17.3 | **Arg138** | 22.4 | **Asp164** | 41.5 |
| **Leu9** | 86.5 | **Trp35** | 0 | **Ile61** | 1.1 | **Val87** | 56.2 | **Tyr113** | 19.7 | **Glu139** | 82.4 | **Leu165** | 0.8 |
| **Tyr10** | 74.1 | **Tyr36** | 23.4 | **Lys62** | 61.1 | **Ala88** | 0.2 | **Asp114** | 87.3 | **Pro140** | 55.3 | **Thr166** | 6.3 |
| **Phe11** | 58.7 | **Ser37** | 2.1 | **Ala63** | 44.8 | **Asp89** | 65.3 | **Glu115** | 50.6 | **Asp141** | 60.7 | **Glu167** | 92.1 |
| **Gln12** | 85.5 | **Ile38** | 0 | **Leu64** | 34 | **Lys90** | 36.6 | **Tyr116** | 1.8 | **Val142** | 2.8 | **Val168** | 48.3 |
| **Gly13** | 75.9 | **Leu39** | 2 | **Asp65** | 59.1 | **Thr91** | 48.5 | **Ile117** | 0.7 | **Ser143** | 57.2 | **Asp169** | 41.8 |
| **His14** | 38.2 | **Leu40** | 0.7 | **Asn66** | 100 | **Lys92** | 70.3 | **Leu118** | 0.9 | **Gln144** | 75.2 | **Arg170** | 4.2 |
| **Met15** | 93 | **Ala41** | 0 | **Ser67** | 54.3 | **Asp93** | 37.2 | **Leu119** | 2.8 | **Lys145** | 95.5 | **Cys171** | 0.1 |
| **His16** | 66.7 | **Ser42** | 0 | **Ser68** | 18.3 | **Gly94** | 6.7 | **His120** | 12.8 | **Leu146** | 9.8 | **Leu172** | 44.9 |
| **Glu17** | 99.5 | **Asp43** | 37 | **Leu69** | 0.4 | **Val95** | 24.1 | **Leu121** | 9.1 | **Lys147** | 18.8 | **Gln173** | 65.5 |
| **Glu18** | 36.4 | **Val44** | 3.7 | **Ser70** | 16.3 | **Tyr96** | 0.3 | **Leu122** | 45.1 | **Glu148** | 68 | **Ala174** | 17.8 |
| **Glu19** | 74.7 | **Lys45** | 60.1 | **Phe71** | 4.1 | **Thr97** | 21 | **Asn123** | 0.1 | **Lys149** | 23.8 | **Arg175** | 14.9 |
| **Asn20** | 80.6 | **Glu46** | 65.2 | **Val72** | 1.7 | **Val98** | 8.1 | **Phe124** | 49.4 | **Phe150** | 0 | **Gly176** | 66.8 |
| **Val21** | 8.1 | **Lys47** | 25.3 | **Phe73** | 7.2 | **Val99** | 80.9 | **Asp125** | 44.8 | **Val151** | 54.6 | **Ser177** | 73.7 |
| **Val22** | 22.7 | **Ile48** | 0.3 | **His74** | 4.6 | **Tyr100** | 24 | **Lys126** | 100 | **Lys152** | 78.4 | **Glu178** | 98.1 |
| **Arg23** | 57.7 | **Glu49** | 47.8 | **Thr75** | 7.5 | **Asp101** | 30.2 | **Thr127** | 94.5 | **Tyr153** | 14.6 | **Val179** | 92.3 |
| **Ser24** | 85.1 | **Glu50** | 87.6 | **Lys76** | 40.5 | **Gly102** | 45.3 | **Arg128** | 64.5 | **Cys154** | 0 | **Ala180** | 100 |
| **Asn25** | 99.5 | **Asn51** | 96.8 | **Glu77** | 56.5 | **Tyr103** | 60.9 | **Pro129** | 65.4 | **Gln155** | 74 |  |  |
| **Ile26** | 1.2 | **Gly52** | 15.3 | **Asn78** | 95.8 | **Asn104** | 0.6 | **Phe130** | 0.6 | **Glu156** | 55 |  |  |
| **Asp27** | 45.3 | **Ser53** | 31.2 | **Gly79** | 72.1 | **Val105** | 25.8 | **Gln131** | 37.6 | **His157** | 40.4 |  |  |
| **Ile28** | 21.7 | **Met54** | 3.3 | **Lys80** | 82.2 | **Phe106** | 4.6 | **Leu132** | 1.1 | **Gly158** | 62.6 |  |  |
| **Ser29** | 83.3 | **Arg55** | 12.2 | **Cys81** | 9.9 | **Ser107** | 46.1 | **Val133** | 0 | **Ile159** | 2.4 |  |  |
| **Lys30** | 64.2 | **Val56** | 6.2 | **Thr82** | 34.3 | **Ile108** | 1.3 | **Glu134** | 5.2 | **Val160** | 71.1 |  |  |
| *Residues with a ratio (%) greater than 50% are considered surface-exposed, while those with a ratio (%) below 20% are classified as buried. | | | | | | | | | | | | | |

## Table S14. The amino acid sequence of Fel d 4-derived peptides.

| **Name of the peptide** | **Position (aa)** | **Amino acid sequence** | **No of amino acids** | **Molecular mass (Da)** | **Isoelectric point** |
| --- | --- | --- | --- | --- | --- |
| P1 | 16-50 | HEEENVVRSNIDISKISGEWYSILLASDVKEKIEE**C** | 36 | 4163.62 | 4.48 |
| P2 | 60-102 | HIKALDNSSLSFVFHTKENGKCTEIFLVADKTKDGVYTVVYDG | 43 | 4791.41 | 6.03 |
| P3 | 120-154 | HLLNFDKTRPFQLVEFYAREPDVSQKLKEKFVKYC | 35 | 4318.02 | 8.94 |
| P4 | 161-186 | NILDLTEVDRCLQARGSEVAQDSSVE | 26 | 2848.09 | 4.02 |

The red mark indicates a cysteine residue added for coupling purposes.

## Table S15. Inhibitions of allergic patients IgE binding to E_rFel d 4 and M_rFel d 4 by anti-Fel d 4 rabbit antibodies.

|  | **OD (rFel d 4)** | | | | | |
| --- | --- | --- | --- | --- | --- | --- |
|  | **E_rFel d 4** | | | **M_rFel d 4** | | |
| **Patient** | **Pre immune** | **Anti-Fel d 4** | **% Inhibition** | **Pre immune** | **Anti-Fel d 4** | **% Inhibition** |
| P1 | 0.52 | 0.17 | **66.6** | 0.50 | 0.17 | **66.4** |
| P2 | 0.40 | 0.11 | **71.8** | 0.44 | 0.12 | **72.0** |
| P3 | 1.38 | 0.22 | **84.3** | 1.61 | 0.20 | **87.5** |
| P4 | 1.55 | 0.14 | **90.7** | 0.81 | 0.12 | **84.7** |
| P5 | 0.67 | 0.14 | **79.3** | 0.66 | 0.14 | **79.2** |
| P6 | 4.13 | 0.50 | **87.8** | 4.26 | 0.50 | **88.2** |
| P10 | 2.15 | 0.68 | **68.5** | 1.54 | 0.47 | **69.7** |
| P11 | 1.53 | 0.54 | **64.6** | 1.97 | 0.58 | **70.8** |
| P13 | 1.12 | 0.66 | **41.1** | 1.60 | 0.63 | **60.8** |
| P14 | 2.20 | 0.70 | **68.0** | 2.90 | 0.61 | **78.8** |
| **Mean** | 1.56 | 0.39 | **72.27** | 1.63 | 0.35 | **75.80** |

## Table S16. Inhibitions of allergic patients IgE binding to E_rFel d 4 and M_rFel d 4 by anti-P4 rabbit antibodies.

|  | **OD (rFel d 4)** | | | | | |
| --- | --- | --- | --- | --- | --- | --- |
|  | **E_rFel d 4** | | | **M_rFel d 4** | | |
| **Patient** | **Pre immune** | **Anti-P4** | **% Inhibition** | **Pre immune** | **Anti-P4** | **% Inhibition** |
| P1 | 0.43 | 0.23 | **46.1** | 0.40 | 0.19 | **52.0** |
| P2 | 0.41 | 0.21 | **48.0** | 0.45 | 0.16 | **64.0** |
| P3 | 1.24 | 0.56 | **55.0** | 1.27 | 0.35 | **72.8** |
| P4 | 1.32 | 0.49 | **63.3** | 0.71 | 0.20 | **71.9** |
| P5 | 0.85 | 0.28 | **67.2** | 0.66 | 0.20 | **69.1** |
| P6 | 4.35 | 2.83 | **34.8** | 4.15 | 1.64 | **60.6** |
| P10 | 2.16 | 0.82 | **61.9** | 1.62 | 0.69 | **57.1** |
| P11 | 1.60 | 0.98 | **38.4** | 1.88 | 1.02 | **45.7** |
| P13 | 1.26 | 0.98 | **21.9** | 1.58 | 0.98 | **37.9** |
| P14 | 1.93 | 0.89 | **53.9** | 2.78 | 1.44 | **48.5** |
| **Mean** | 1.55 | 0.83 | **49.04** | 1.55 | 0.69 | **57.95** |

##
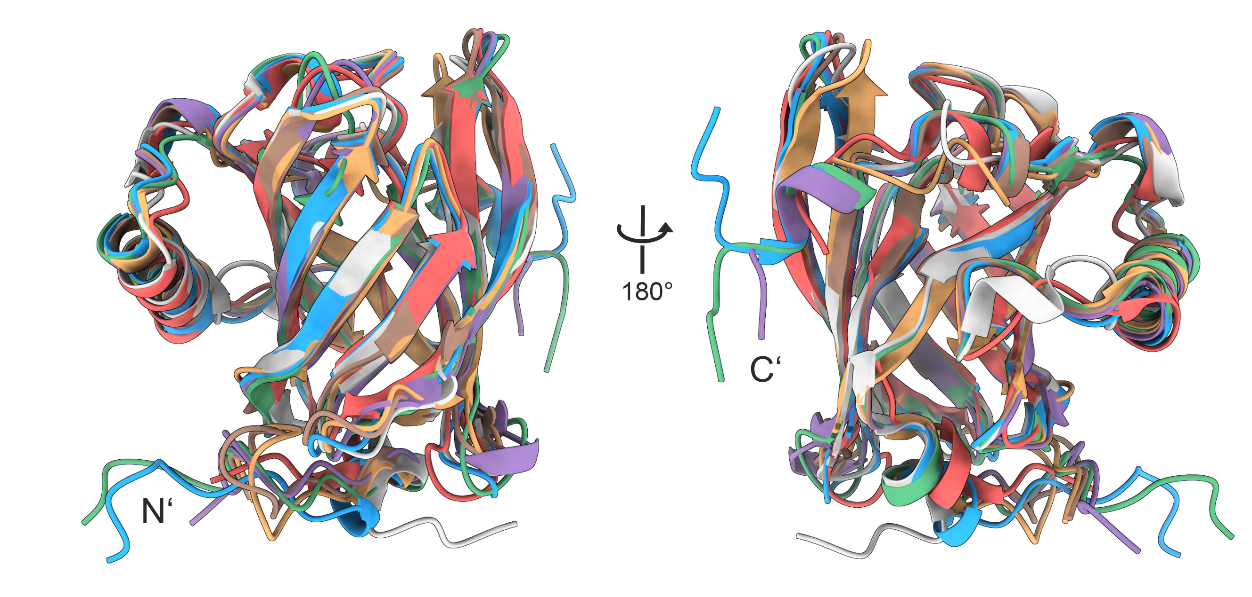
Figure S5. Superimposed 3D structures of selected lipocalin allergens.

Structural alignment in two orientations of E_rFel d 4 (green), Ory c 4 (blue, AlphaFold2 model [8]), Cav p 6 (purple, AlphaFold2 model [8]), Rat n 1 (brown, PDB: 2A2G), Mus m 1 (orange, PDB: 1MUP), Bos d 2 (red, PDB: 4WFU) and Can f 2 (grey, PDB: 3L4R). N-terminal extension (11 N-terminal residues) in E_rFel d 4 structure model was not shown for simplicity, (N’- N-terminus, C’- C-terminus).

## Table S17. RMSD values of alignment for selected lipocalin homologues.

| **E_rFel d 4 (PDB: 9I2M)** | **Number of aligned Cα atoms** | **RMSD (Å)** |
| --- | --- | --- |
| **Can f 6** (PDB: 6NRE) | 152/156 | 0.647 |
| **Equ c 1** (PDB: 1EW3) | 138/156 | 0.783 |
| **Ory c 4** (AlphaFold2 model) | 138/169 | 0.707 |
| **Cav p 6** (AlphaFold2 model) | 154/161 | 0.678 |
| **Rat n 1** (PDB: 2A2G) | 138/155 | 0.750 |
| **Mus m 1** (PDB: 1MUP) | 129/154 | 0.720 |
| **Bos d 2** (PDB: 4WFU) | 116/148 | 0.988 |
| **Can f 2** (PDB: 3L4R) | 119/148 | 0.782 |

Structural alignment for selected lipocalin allergens. Shown is number of aligned atoms and the Cα atom root-mean-square deviation (RMSD) values calculated for homologous lipocalins. Cα atoms with more than 2 Å distance were pruned by default.

## Figure S6. Sequence alignment of selected homologous lipocalin allergens.


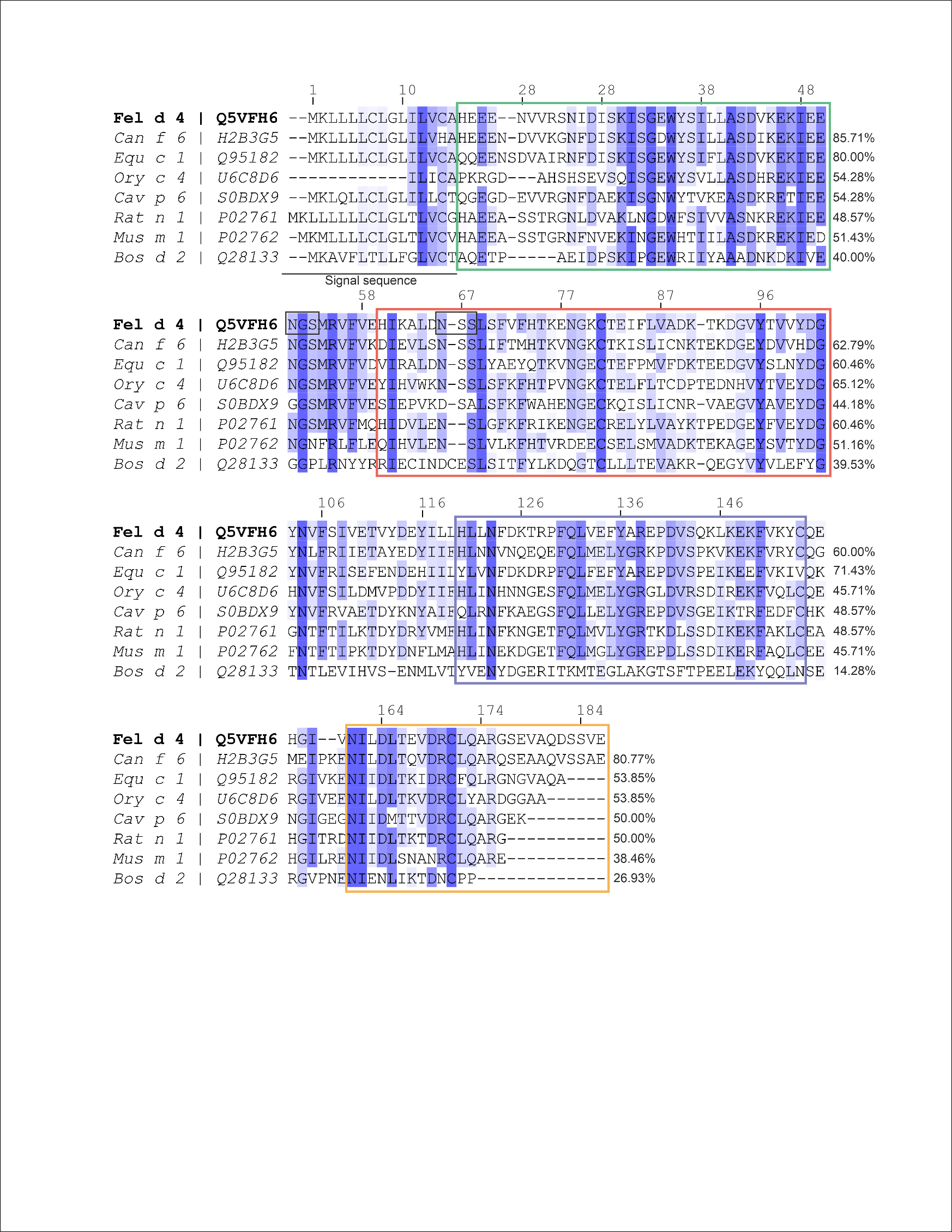


Sequence alignment of Fel d 4 and homologous lipocalin allergens from other animals generated using Clustal Omega [9] and displayed with JalView 2.11.3.2 under standard settings [10]. Residues were colored from blue to white according to percent identity decrease, with the 20% conservation threshold. Peptides 1, 2, 3, and 4 are boxed and indicated in different colors. Box of three amino acids (black) indicate N-glycosylation residues predicted by NetNGlyc 1.0 [11]. The percentage identity of Fel d 4 peptides towards corresponding sequences in other lipocalins are shown on the right. Nomenclature of the allergens is according to WHO/IUIS Allergen Nomenclature Sub-Committee ([allergen.org](http://www.allergen.org)): Fel d - *Felis domesticus*, Can f - *Canis familiaris*, Equ c - *Equus caballus*, Oru c - *Oryctolagus cuniculus*, Cav p - *Cavia porcellus*, Rat n 1 - *Rattus norvegicus*, Mus m - *Mus musculus*, Bos d - *Bos domesticus*.

## Table S18. Inhibitions of allergic patients IgE binding to rEqu c 1 by anti-Fel d 4 and anti-P4 rabbit antibodies.

|  | **OD (rEqu c 1)** | | | | | |
| --- | --- | --- | --- | --- | --- | --- |
| **Patient** | **Pre immune** | **Anti-Fel d 4** | **% Inhibition** | **Pre immune** | **Anti-P4** | **% Inhibition** |
| P13 | 0.39 | 0.063 | 83.82 | 0.43 | 0.26 | 40.5 |
| P9 | 1.43 | 0.26 | 80.7 | 1.08 | 1.02 | 5.3 |
| P21 | 1.57 | 0.18 | 88.6 | 1.14 | 0.61 | 46.3 |
| P11 | 1.06 | 0.12 | 88.6 | 0.71 | 0.43 | 38.7 |
| **Mean** | 1.11 | 0.16 | **85.4** | 0.84 | 0.58 | **32.7** |

##

## Table S19. Demographic, clinical, and serological characteristics of cat allergic patients.

| **Patient** | **Age** | **Sex** | **Cat allergy symptoms** | | | | **Other Allergies** | **Specific IgE (e1) kUa/L** | **Total IgE kU/L** | **Fel d 4**  **(kUA/L)** | **Can f 6**  **(ISU-IgE)** | **Equ c 1**  **(ISU-IgE)** |
| --- | --- | --- | --- | --- | --- | --- | --- | --- | --- | --- | --- | --- |
|  |  |  | **Asth** | **Rh** | **Con** | **AD** |  |  |  |  |  |  |
| 1 | 27 | m | - | + | + | - | p, hdm, nu | 20.8 | 938 | 7.38 | 0 | 0 |
| 2 | 31 | f | + | + | + | - | p | 52.7 | >5000 | 11.3 | 0 | 0 |
| 3 | 26 | m | + | + | - | + | p, hdm | nd | nd | 1 | nd | nd |
| 4 | 31 | m | - | + | + | - | drug | 11.3 | 101 | 6.95 | 0 | 0 |
| 5 | 29 | f | + | + | + | - | p, hdm | >100 | >2000 | >100 | 8 | 11.1 |
| 6 | 40 | f | + | + | + | + | p, hdm, nu, fi | >100 | 570 | 1.11 | 0 | 0 |
| 7 | 26 | f | + | + | + | + | p | nd | nd | 0.44 | 0 | 0 |
| 8 | 49 | f | + | + | + | - | hdm | nd | nd | 3.9 | 0 | 1.31 |
| 9 | 29 | f | + | + | + | - | p, hdm | >100 | 1224 | 1.07 | 4.2 | 5.8 |
| 10 | 47 | f | + | - | - | + | p | >100 | >5000 | 22 | 0.5 | 25 |
| 11 | 31 | f | - | - | - | + | p, hdm | 15.7 | 218 | 5.27 | 0 | 3 |
| 12 | 36 | f | + | + | + |  | p, hdm | nd | nd | 9 | 0 | 17 |
| 13 | 29 | f | - | + | + | - | p, hdm | 78.1 | >2000 | 7.38 | 0 | 0.28 |
| 14 | 39 | m | + | - | - | + | p, hdm | nd | nd | 1.67 | nd | nd |
| 15 | 41 | f | + | - | - | - | p, hdm | nd | nd | 3 |  | 1.26 |
| 16 | 21 | m | - | + | + | - | p, hdm | nd | nd | 2.64 | 0 | 3.02 |
| 17 | 22 | f | - | + | + | + | p, hdm | nd | nd | 3.06 | 0 | 7.34 |
| 18 | 22 | m | - | + | + | - | p, hdm | 3.6 | 141 | 2.64 | 0 | 0 |
| 19 | 37 | m | + | + | + | + | p, hdm | 46.8 | 290 | 2.47 | 0 | 0 |
| 20 | 40 | m | + | + | + | - | p, hdm, nu | 20.8 | 938 | 4.16 | 0 | 0 |
| 21 | 29 | m | + | + | + | + | P, hdm | 19.7 | >2000 | 3.42 | 0 | 4.5 |
| NA | 27 | f | - | - | - | - | no | nd | nd | 0 | 0 | 0 |

Demographic data, clinical symptoms, and sensitization to other allergen sources are displayed for cat-allergic patients and non-allergic. Total and Fel d 4-specific IgE levels were measured by using ImmunoCAP and are displayed in kilo units/liter (kU/l) and kilo units of antigen/liter (kUA/l), respectively, Can f 6- and Equ c 1-specific IgE levels were measured by using ImmunoCAP ISAC and displayed in ISAC Standardized Units (ISU-E). The cutoff value is 0.1 kUA/l.

Abbreviations: M, male; F, female; Asth, asthma; Rh, rhinitis; Con, conjunctivitis; AD, atopic dermatitis; e1, cat dander extract; nd, not determined; p, pollen; hdm, house dust mite; nu, nuts; fi, fish.

**
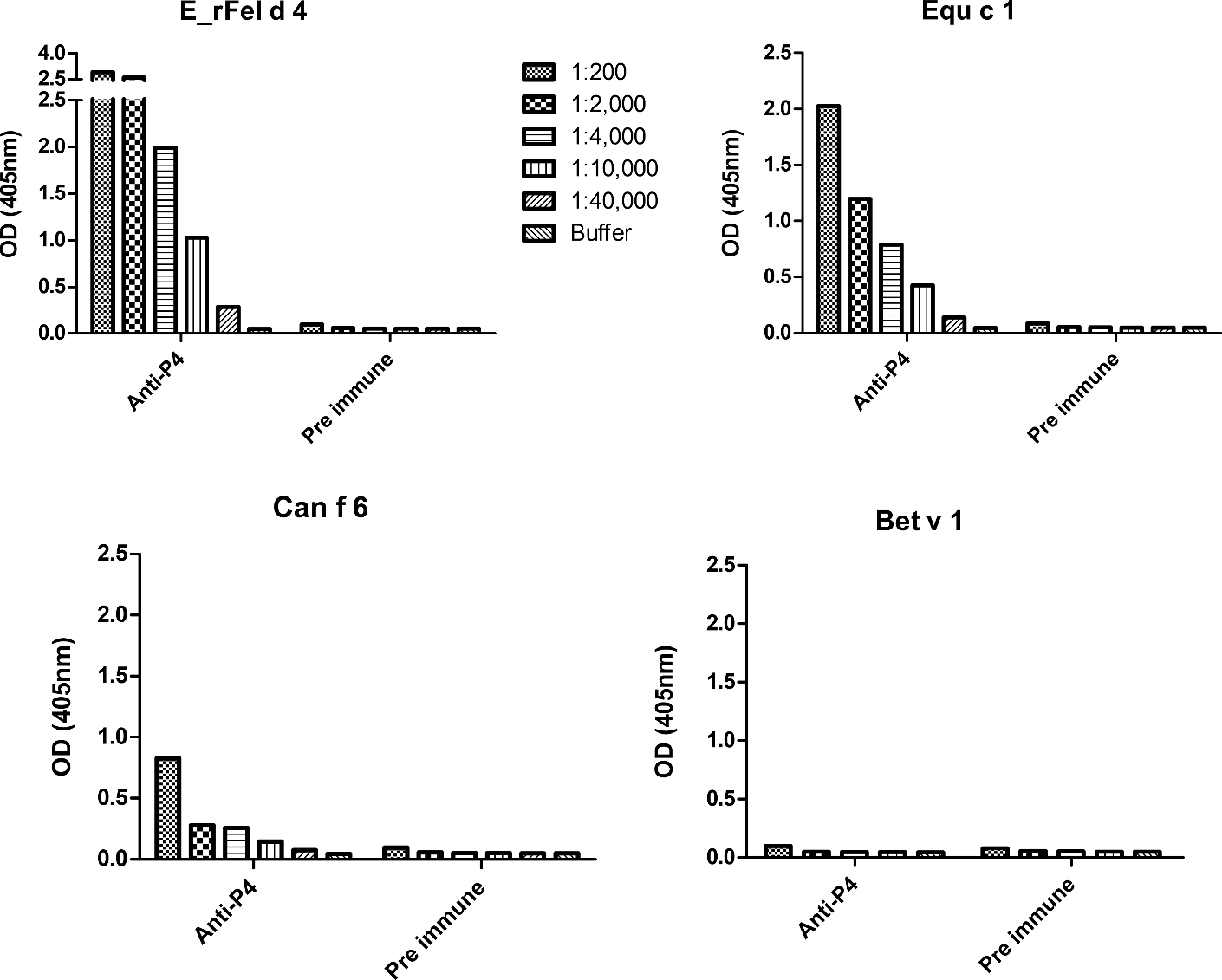
Figure S7. Cross-reactivity of rabbit anti-P4 antibodies with rEqu c 1 and rCan f 6.**

IgG reactivity (y-axes: Average OD values 405/492 nm corresponding to levels of bound specific IgG antibodies) of different dilutions (x-axes) of rabbit anti-P4 antisera (left) or the corresponding pre-immune sera (right) with E_rFel d 4, rEqu c 1, rCan f 6 or rBet v 1.

## Table S20. Cross-reactivity of rabbit anti-P4 antibodies with rEqu c 1 and rCan f 6.

|  | **Dilution** | **E_rFel d 4** | **rEqu c 1** | **rCan f 6** | **rBet v 1** |
| --- | --- | --- | --- | --- | --- |
| **Anti-P4** | 1:200 | 2.906 | 2.025 | 0.829 | 0.098 |
|  | 1:2,000 | 2.588 | 1.197 | 0.278 | 0.049 |
|  | 1:4,000 | 1.991 | 0.788 | 0.257 | 0.048 |
|  | 1:10,000 | 1.025 | 0.427 | 0.147 | 0.046 |
|  | 1:40,000 | 0.284 | 0.140 | 0.077 | \ |
|  | Buffer | 0.048 | 0.048 | 0.046 | 0.044 |
| **Pre-immune** | 1:200 | 0.097 | 0.086 | 0.097 | 0.080 |
|  | 1:2,000 | 0.060 | 0.055 | 0.058 | 0.055 |
|  | 1:4,000 | 0.054 | 0.051 | 0.054 | 0.054 |
|  | 1:10,000 | 0.052 | 0.049 | 0.051 | 0.050 |
|  | 1:40,000 | 0.051 | 0.048 | 0.049 | \ |
|  | Buffer | 0.051 | 0.048 | 0.049 | 0.049 |

# Supplementary References

1. Micsonai A, Moussong É, Wien F, et al. BeStSel: webserver for secondary structure and fold prediction for protein CD spectroscopy. *Nucleic Acids Research*. 2022;50(W1):W90-W98. doi:10.1093/nar/gkac345

2. Flot D, Mairs T, Giraud T, et al. The ID23-2 structural biology microfocus beamline at the ESRF. *J Synchrotron Radiat*. 2010;17(Pt 1):107-118. doi:10.1107/S0909049509041168

3. Burkhardt A, Pakendorf T, Reime B, et al. Status of the crystallography beamlines at PETRA III. *Eur Phys J Plus*. 2016;131(3):56. doi:10.1140/epjp/i2016-16056-0

4. Kabsch W. XDS. *Acta Crystallogr D Biol Crystallogr*. 2010;66(Pt 2):125-132. doi:10.1107/S0907444909047337

5. Evans PR, Murshudov GN. How good are my data and what is the resolution? *Acta Crystallogr D Biol Crystallogr*. 2013;69(Pt 7):1204-1214. doi:10.1107/S0907444913000061

6. Trifonova D, Curin M, Riabova K, et al. Allergenic Activity of Individual Cat Allergen Molecules. *International Journal of Molecular Sciences*. 2023;24(23):16729. doi:10.3390/ijms242316729

7. Focke M, Mahler V, Ball T, et al. Nonanaphylactic synthetic peptides derived from B cell epitopes of the major grass pollen allergen, Phl p 1, for allergy vaccination. *FASEB J*. 2001;15(11):2042-2044. doi:10.1096/fj.01-0016fje

8. Jumper J, Evans R, Pritzel A, et al. Highly accurate protein structure prediction with AlphaFold. *Nature*. 2021;596(7873):583-589. doi:10.1038/s41586-021-03819-2

9. Madeira F, Madhusoodanan N, Lee J, et al. The EMBL-EBI Job Dispatcher sequence analysis tools framework in 2024. *Nucleic Acids Res*. 2024;52(W1):W521-W525. doi:10.1093/nar/gkae241

10. Waterhouse AM, Procter JB, Martin DMA, Clamp M, Barton GJ. Jalview Version 2--a multiple sequence alignment editor and analysis workbench. *Bioinformatics*. 2009;25(9):1189-1191. doi:10.1093/bioinformatics/btp033

11. Gupta R, Brunak S. Prediction of glycosylation across the human proteome and the correlation to protein function. *Pac Symp Biocomput*. Published online 2002:310-322.
